# Acetylation of Oleanolic Acid Dimers as a Method of Synthesis of Powerful Cytotoxic Agents

**DOI:** 10.3390/molecules29184291

**Published:** 2024-09-10

**Authors:** Andrzej Günther, Przemysław Zalewski, Szymon Sip, Piotr Ruszkowski, Barbara Bednarczyk-Cwynar

**Affiliations:** 1 Department of Organic Chemistry, Faculty of Pharmacy, Poznan University of Medical Sciences, Collegium Pharmaceuticum 2 (CP.2), Rokietnicka Str. 3, 60-806 Poznan, Poland; bcwynar@ump.edu.pl; 2 Department of Pharmacognosy and Biomaterials, Faculty of Pharmacy, Poznan University of Medical Sciences, Collegium Pharmaceuticum 1 (CP.1), Rokietnicka Str. 3, 60-806 Poznan, Poland; pzalewski@ump.edu.pl (P.Z.); szymonsip@ump.edu.pl (S.S.); 3 Department of Pharmacology, Faculty of Pharmacy, Poznan University of Medical Sciences, Collegium Pharmaceuticum 1 (CP.1), Rokietnicka Str. 3, 60-806 Poznan, Poland; pruszkowski@gmail.com; 4 Center of Innovative Pharmaceutical Technology (CITF), Rokietnicka Str. 3, 60-806 Poznan, Poland

**Keywords:** oleanolic acid, triterpene dimers, oleanolic acid dimers, dimerization, acetylation, chemical modification of triterpenes, chemical modification of natural compounds, SAR, cytotoxic activity, antioxidant activity

## Abstract

Oleanolic acid, a naturally occurring triterpenoid compound, has garnered significant attention in the scientific community due to its diverse pharmacological properties. Continuing our previous work on the synthesis of oleanolic acid dimers (OADs), a simple, economical, and safe acetylation reaction was performed. The newly obtained derivatives (AcOADs, **3a**–**3n**) were purified using two methods. The structures of all acetylated dimers (**3a**–**3n**) were determined based on spectral methods (IR, NMR). For all AcOADs (**3a**–**3n**), the relationship between the structure and the expected directions of pharmacological activity was determined using a computational method (QSAR computational analysis). All dimers were also tested for their cytotoxic activity on the SKBR-3, SKOV-3, PC-3, and U-87 cancer cell lines. HDF cell line was applied to evaluate the Selectivity Index of the tested compounds. All cytotoxic tests were performed with the application of the MTT assay. Finally, all dimers of oleanolic acid were subjected to DPPH and CUPRAC tests to evaluate their antioxidant activity. The obtained results indicate a very high level of cytotoxic activity (IC_50_ for most AcOADs below 5.00 µM) and a fairly high level of antioxidant activity (Trolox equivalent in some cases above 0.04 mg/mL).

## 1. Introduction

**Figure 1:**
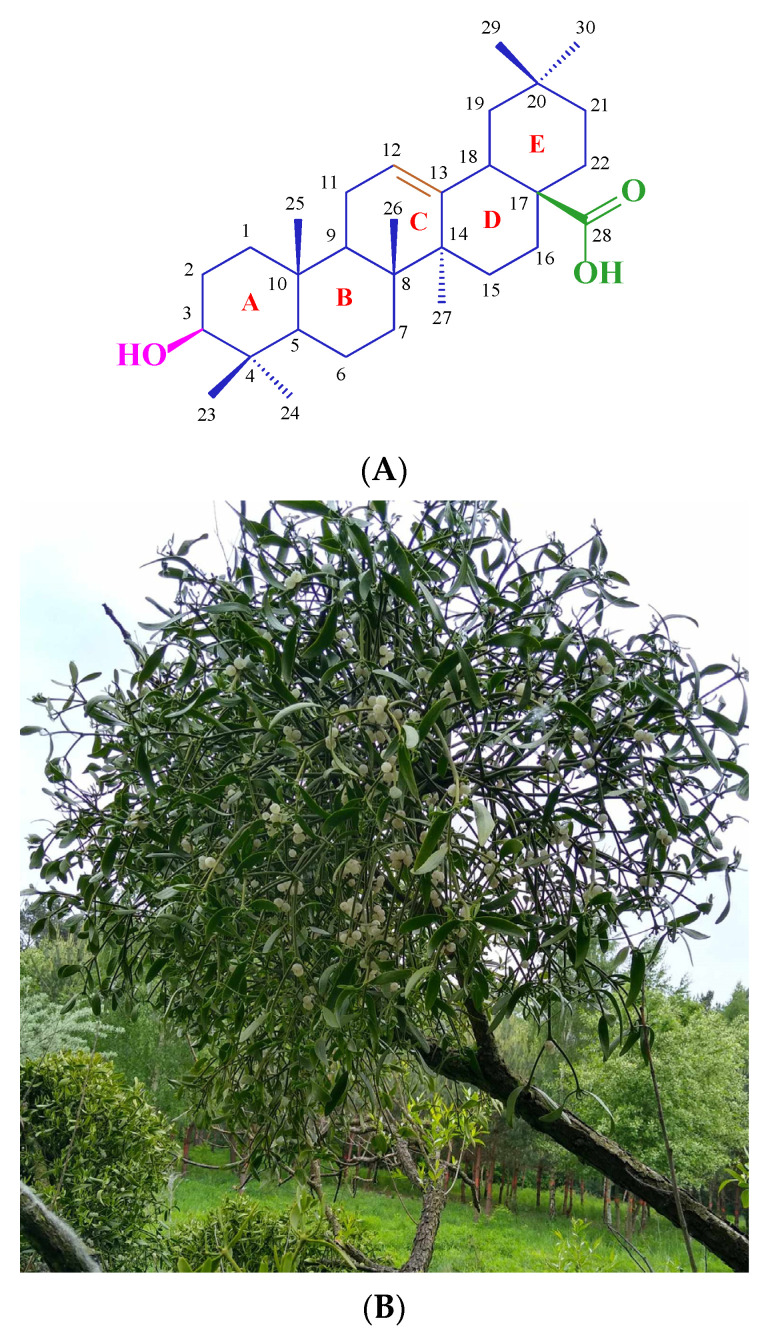
(**A**) Structure of oleanolic acid (**1**) and (**B**) one of the sources of this compound—mistletoe (*Viscum alba*) herb.

Triterpenoids, also known as isoprenoids, are a vast group of plant secondary metabolites, including at least 40,000 species [1]. These substances occur in plants as glycosides, or as aglycones, and, depending on their structure, they can be divided into monoterpenoids, diterpenoids, sesquiterpenoids, triterpenoids, etc. The most numerous and most widespread group in the plant world are triterpenoids, compounds containing 30 carbon atoms that create various basic skeletons. The structure of these carbon skeletons is the criterion for dividing triterpenoids into several subgroups, the most important of which are oleananes, ursanes, lupanes, and friedelanes. Oleananes, the first of the mentioned, is the most popular and numerous group of compounds among the triterpenes. Oleanolic acid (Figure 1A) is the representative of this group and is the parent compound for the synthesis of all derivatives presented in this publication. So far, the presence of oleanolic acid has been proven in at least 1600 species of medicinal and edible plants [2]. Some of the richest sources of oleanolic acid include olive leaves and fruit (*Olea europaea*) [3], marigold herb (*Calendula officinalis*) [4], and mistletoe herb (*Viscum alba*, Figure 1B) [5].

Oleanolic acid is a valuable substrate in the synthesis of numerous derivatives with expected pharmacological activity. The second reason it is valued among scientists is its numerous and valuable pharmacological properties. The latest research has shown, among others, antidiabetic [6], neuroprotective [7], antioxidative and antiglycative [8], antiaging [9], antidermatitic [10], osteoprotective [11], anti-inflammatory [12], antinociceptive [13], gastroprotective [14], liver-protective [15], antiviral [16], anticancer [17] and other activities.

The greatest hopes regarding various directions of pharmacological activity of oleanolic acid (**1**) are associated with broadly understood “anticancer” activity, including antitumor, cytotoxic against cancer cells, cytostatic against cancer cells, etc. activities. Cancer, after cardiovascular disease, is the second cause of death in the world. All reports regarding the number of cancer cases and the number of deaths caused by them indicate upward trends [18], so it is really important to search for new, effective, and selective anticancer agents. Studies published in recent years indicate high anticancer effectiveness of oleanolic acid (**1**) against e.g., breast cancer [19], melanoma [20], colon carcinoma [21], hepatoma [22], cervical carcinoma [23], osteosarcoma [24] and other carcinomas.

Cancer diseases, as well as many other diseases, e.g., autoimmune disorders, aging, cataracts, rheumatoid arthritis, and cardiovascular and neurodegenerative diseases, are caused by, among others, oxidative stress [25]. It is caused by excessive production of Reactive Oxygen Species (ROS, free radicals) or their improper use by the body [26]. Under normal physiological conditions, free radicals exert beneficial effects on cellular responses and immune function [25]. At high concentrations, they generate oxidative stress, a deleterious process that can damage cell structures, such as DNA, lipids, and proteins [27].

The antioxidant effect of oleanolic acid (**1**) probably involves quenching ROS, inhibiting lipid peroxidation, or indirectly stimulating cellular antioxidant defenses [28]. Antioxidant and free radicals scavenging activity of triterpenes are the subject of many works, e.g., [29,30].

As we mentioned earlier, oleanolic acid (**1**), a naturally occurring triterpenoid compound, has garnered significant attention in the scientific community due to its diverse pharmacological properties, including anticancer and antioxidant activities. Recent studies have focused on exploring structural modifications at the C-17 position of oleanolic acid (**1**) in an effort to further enhance different biological activities.

SAR studies showed that derivatives of oleanolic acid (**1**) with structural modifications made within the C-17 carboxyl group, either forming esters or amides, significantly increased their antitumor activity [31,32]. The diamine-PEGylated oleanolic acid derivative (of amide structure) has potent anti-inflammatory activity in both in vitro and in vivo models [33]. The acetylated derivative of oleanolic acid methyl ester showed an anti-inflammatory effect in serotonin- and fresh egg-albumin-activated inflammatory models in male Wistar rats [34]. Another derivative, oleanolic acid amide with 1,6-hexanediamine moiety, showed a significant antimicrobial effect in vitro and decreased the toxicity of the parent compound (**1**) by decreasing MIC in most of the tested Gram-positive bacteria, highlighting its effectiveness against *Staphylococcus aureus* and methicillin-resistant *Staphylococcus aureus* (MRSA) [35]. Oleanolic acid esters containing unsaturated bonds, when compared to the mother compound (**1**), were effective anticancer agents [36]. Other esters of this triterpene, containing uridine [37] or triazole system [38,39], were also efficient anticancer agents and demonstrated excellent inhibition of proliferation when compared to the starting compound (**1**). Oleanolic acid conjugates with cyclodextrines showed the most promising antiviral effects without associated toxicity against host cells, thus suggesting the occurrence of high antiviral selectivity that should be further investigated [40].

The above examples show that chemical modifications of the carboxyl group at the C-17 position of the oleanolic acid molecule (**1**) are an excellent way to obtain derivatives with a high level of pharmacological activity. They also confirm that our topic of chemical modification of the oleanolic acid (**1**) molecule by dimerization using the C-17 carboxyl group is an extremely future trend that will result in obtaining numerous derivatives with an interesting structure and valuable utilitarian properties, including pharmacological ones.

Triterpene dimers are an extremely interesting and almost unexplored research topic. Joining two molecules of oleanolic acid through its C-17 carboxyl group is an excellent example of a reaction in which appropriately selected parameters determine the profitability of obtaining products.

Apart from our previous works [41,42], only one work is known from the scientific literature, which presented an initially developed procedure for the dimerization of oleanolic acid through its C-17 carboxyl group [43]. With this method, after laborious purification on a silica gel column, only a few dimers with an even number of carbon atoms in the saturated liker have been obtained. These compounds showed a high level of cytostatic activity. In some cases, the IC_50_ value did not exceed 10 µM [43]. These results encouraged us to modify the known method and use it to obtain not only a set of dimers containing from 1 to as many as 12 carbon atoms in the linker but also a linker containing a *cis*- or *trans*-unsaturated bond.

While our previous studies have examined the method of OADs synthesis, their physicochemical properties, SAR analysis, cytotoxic activity, SI, and antioxidant activity developed with DPPH and CUPRAC assays, there remains a significant gap in understanding, e.g., physicochemical properties, SAR analysis, cytotoxic activity, SI and antioxidant activity of modified OADs (e.g., AcOADs). This study aims to address this gap by spectral data analysis and elucidation of how the presence of the acetyl group and the length of the bridge connecting two triterpene units in OADs influence the physicochemical properties of new products and their anticancer and antioxidant activity.

By achieving these objectives, the study aims to provide insights into designing more effective OADs for therapeutic applications, particularly in areas where antioxidant and/or cancer properties are desired.

## 2. Results

### 2.1. Synthesis of Acetylated Oleanolic Acid Dimers (AcOADs) ***3a**–**3n***

**Figure 2:**
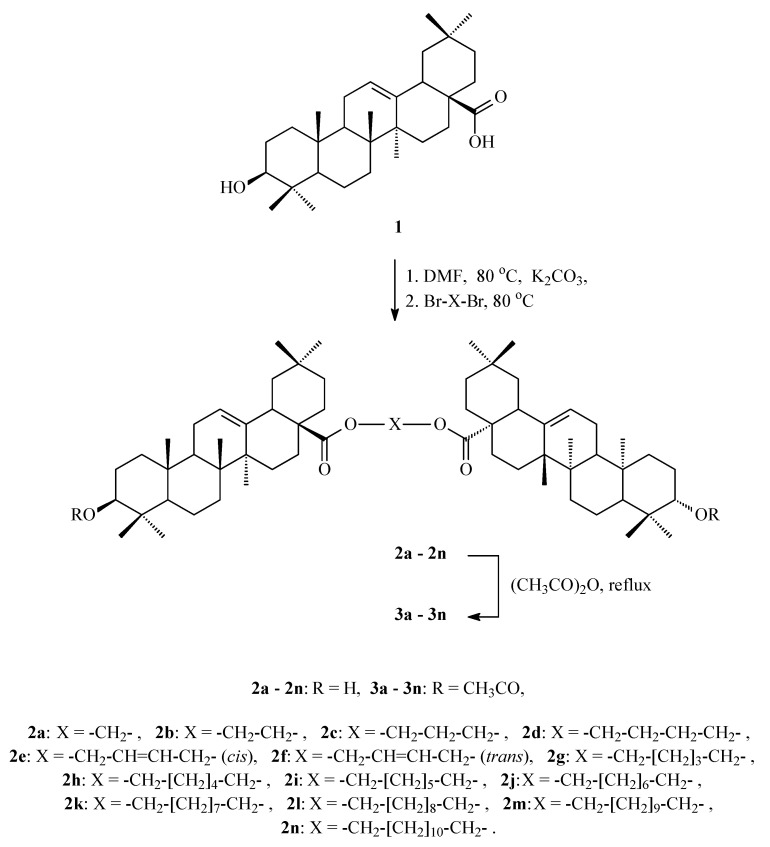
Synthesis of Acetylated Oleanolic Acid Dimers (AcOADs) **3a**–**3n**.

Chemical transformations of oleanolic acid (**1**) leading to AcOADs **3a**–**3n** are shown in Figure 2.

Experimental work covered the acetylation reaction of the previously obtained dimers of oleanolic acid **2a**–**2n** [41]. As a result of this chemical transformation, the Acetylated Oleanolic Acid Dimers (AcOADs) **3a**–**3n** (Figure 2) were obtained with very high efficiency.

### 2.2. Structure–Activity Analysis for Acetylated Oleanolic Acid Dimers (AcOADs) ***3a**–**3n***

The highest results of SAR analysis for Acetylated Oleanolic Acid Dimers (AcOADs) **3a**–**3n** are given in Table 1 and Table 2. The detailed results of the SAR analysis with P_a_ ≥ 0.700 are given in Supplementary Materials (Supplementary Materials S01, Table S1).

### 2.3. Cytotoxic Activity of Acetylated Oleanolic Acid Dimers (AcOADs) ***3a**–**3n***

#### 2.3.1. In Vitro Assay

The cytotoxic activity of oleanolic acid (mother compound, **1**, Figure 2) and its 14 derivatives (**3a**–**3n**, Figure 2) was tested in vitro using the standard MTT method based on viability assay, e.g., [44] against carcinoma cell lines: SKBR-3, SKOV-3, PC-3, U-87, and one normal cell line: HDF. The estimated IC_50_ values for compounds **1** and **3a**–**3n** are presented in Table 3.

**Table 1:** The highest predicted activity of oleanolic acid (**1**) and Acetylated Oleanolic Acid Dimers (AcOADs) **3a**–**3g** determined by the PASS method [45].

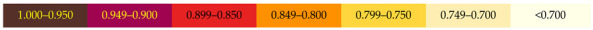								

**Activity**	**P_a_ Factor (and P_i_ Factor) of Compounds: 1 and 3a–3g**							
	**OA (1)**	**3a**	**3b**	**3c**	**3d**	**3e**	**3f**	**3g**
**Antiprotozoal** **(Leishmania)**	0.721 (0.008)	0.820 (0.004)	0.862 (0.004)	0.892 (0.003)	0.891 (0.003)	0.854 (0.004)	0.854 (0.004)	0.904 (0.003)
**Apoptosis agonist**	0.901 (0.004)	0.905 (0.004)	0.881 (0.05)	0.866 (0.005)	0.868 (0.005)	0.874 (0.005)	0.874 (0.005)	0.866 (0.005)
**Caspase 3 stimulant**	0.984 (0.002)	0.904 (0.003)	0.950 (0.003)	0.909 (0.003)	0.909 (0.003)	0.902 (0.003)	0.902 (0.003)	0.910 (0.003)
**Caspase 8 stimulant**	0.914 (0.001)	0.861 (0.001)	0.889 (0.001)	0.873 (0.001)	0.873 (0.001)	0.852 (0.001)	0.852 (0.001)	0.879 (0.001)
**Chemopreventive**	0.937 (0.002)	0.915 (0.002)	0.896 (0.002)	0.881 (0.003)	0.888 (0.003)	0.928 (0.002)	0.928 (0.002)	0.928 (0.002)
**Hepatoprotectant**	0.930 (0.002)	0.915 (0.002)	0.939 (0.002)	0.941 (0.002)	0.958 (0.001)	0.959 (0.001)	0.959 (0.001)	0.963 (0.001)
**Insulin promotor**	0.869 (0.004)	0.881 (0.003)	0.941 (0.002)	0.928 (0.002)	0.928 (0.002)	0.920 (0.003)	0.920 (0.003)	0.922 (0.002)
**Lipid metabolism** **regulator**	<0.700	0.822 (0.005)	0.959 (0.002)	0.949 (0.003)	0.959 (0.002)	0.960 (0.002)	0.960 (0.002)	0.963 (0.002)
**Membrane integrity** **antagonist**	0.928 (0.002)	0.895 (0.003)	0.936 (0.001)	0.930 (0.002)	0.939 (0.001)	0.913 (0.002)	0.913 (0.002)	0.943 (0.001)
**Oxidoreductase inhibitor**	0.904 (0.002)	0.875 (0.003)	0.904 (0.002)	0.897 (0.002)	0.904 (0.002)	0.913 (0.002)	0.913 (0.002)	0.907 (0.002)
**Transcription factor NF kappa B stimulant**	0.954 (0.001)	0.916 (0.001)	0.918 (0.001)	0.911 (0.001)	0.911 (0.001)	0.911 (0.001)	0.911 (0.001)	0.908 (0.001)
**Transcription factor** **stimulant**	0.954 (0.001)	0.916 (0.001)	0.918 (0.001)	0.911 (0.001)	0.911 (0.001)	0.911 (0.001)	0.911 (0.001)	0.908 (0.001)

**Legend**: **P_a_**—probability of activity; **P_i_**—probability of inactivity.

**Table 2:** The highest predicted activity of Acetylated Oleanolic Acid Dimers (AcOADs) **3h**–**3n** determined by the PASS method [45].

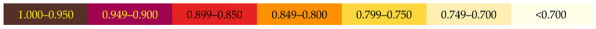							

**Activity**	**P_a_ Factor (and P_i_ Factor) of Compounds 3h–3n**						
	**3h**	**3i**	**3j**	**3k**	**3l**	**3m**	**3n**
**Antiprotozoal (Leishmania)**	0.904 (0.003)	0.904 (0.003)	0.904 (0.003)	0.904 (0.003)	0.904 (0.003)	0.904 (0.003)	0.904 (0.003)
**Apoptosis agonist**	0.866 (0.005)	0.866 (0.005)	0.866 (0.005)	0.866 (0.005)	0.866 (0.005)	0.866 (0.005)	0.866 (0.005)
**Caspase 3 stimulant**	0.910 (0.003)	0.910 (0.003)	0.910 (0.003)	0.910 (0.003)	0.910 (0.003)	0.910 (0.003)	0.910 (0.003)
**Caspase 8 stimulant**	0.879 (0.001)	0.879 (0.001)	0.879 (0.001)	0.879 (0.001)	0.879 (0.001)	0.879 (0.001)	0.879 (0.001)
**Chemopreventive**	0.888 (0.003)	0.888 (0.003)	0.888 (0.003)	0.888 (0.003)	0.888 (0.003)	0.888 (0.003)	0.888 (0.003)
**Hepatoprotectant**	0.963 (0.001)	0.963 (0.001)	0.963 (0.001)	0.963 (0.001)	0.963 (0.001)	0.963 (0.001)	0.963 (0.001)
**Insulin promotor**	0.922 (0.002)	0.922 (0.002)	0.922 (0.002)	0.922 (0.002)	0.922 (0.002)	0.922 (0.002)	0.922 (0.002)
**Lipid metabolism regulator**	0.963 (0.002)	0.963 (0.002)	0.963 (0.002)	0.963 (0.002)	0.963 (0.002)	0.963 (0.002)	0.963 (0.002)
**Membrane integrity** **antagonist**	0.943 (0.001)	0.943 (0.001)	0.943 (0.001)	0.943 (0.001)	0.943 (0.001)	0.943 (0.001)	0.943 (0.001)
**Oxidoreductase inhibitor**	0.907 (0.002)	0.907 (0.002)	0.907 (0.002)	0.907 (0.002)	0.907 (0.002)	0.907 (0.002)	0.907 (0.002)
**Transcription factor NF kappa B stimulant**	0.908 (0.001)	0.908 (0.001)	0.908 (0.001)	0.908 (0.001)	0.908 (0.001)	0.908 (0.001)	0.908 (0.001)
**Transcription factor** **stimulant**	0.908 (0.001)	0.908 (0.001)	0.908 (0.001)	0.908 (0.001)	0.908 (0.001)	0.908 (0.001)	0.908 (0.001)

**Legend**: **P_a_**—probability of activity; **P_i_**—probability of inactivity.

**Table 3:** Cytotoxic Activity (IC_50_) and Selectivity Index (SI) of oleanolic acid (**1**) and Acetylated Oleanolic Acid Dimers (AcOADs) **3a**–**3n** against the tested cell lines determined in the MTT assay [44].

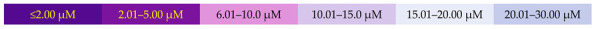										

**Number** **of C Atoms** **in a Linker**	**Comp. No.**	**SKBR-3**		**SKOV-3**		**B**		**U-87**		**HDF**
		**IC_50_ (SD), μM**	**SI**	**IC_50_ (SD), μM**	**SI**	**IC_50_ (SD), μM**	**SI**	**IC_50_ (SD), μM**	**SI**	**IC_50_ (SD), μM**
**0**	**1 (OA)**	19.62 (0.02)	1.27	18.81 (0.09)	1.32	18.63 (0.05)	1.33	18.15 (0.01)	1.37	24.87 (0.04)
**1**	**3a**	2.04 (0.14)	**3.25**	2.29 (0.02)	**2.89**	2.26 (0.03)	**2.93**	2.03 (0.03)	**3.27**	6.63 (0.01)
**2 sat.**	**3b**	2.97 (0.03)	**2.37**	3.04 (0.09)	**2.32**	3.12 (0.04)	**2.26**	3.10 (0.02)	**2.27**	7.05 (0.18)
**3 sat.**	**3c**	3.82 (0.05)	0.80	3.15 (0.09)	0.97	2.94 (0.02)	1.04	3.11 (0.01)	0.99	3.07 (0.12)
**4 sat.**	**3d**	3.03 (0.17)	1.58	2.89 (0.06)	1.65	3.59 (0.02)	1.33	3.54 (0.09)	1.35	4.78 (0.05)
**4 unsat. *cis***	**3e**	3.07 (0.01)	**2.17**	2.95 (0.07)	**2.26**	3.35 (0.04)	1.99	3.48 (0.03)	1.91	6.66 (0.02)
**4 unsat. *trans***	**3f**	7.06 (0.19)	0.57	6.92 (0.03)	0.58	6.70 (0.21)	0.60	6.80 (0.02)	0.59	4.02 (0.01)
**5 sat.**	**3g**	4.65 (0.16)	1.22	4.23 (0.09)	1.34	4.10 (0.08)	1.38	4.09 (0.05)	1.38	5.66 (0.41)
**6 sat.**	**3h**	5.25 (0.01)	1.30	4.72 (0.07)	1.45	5.13 (0.11)	1.33	4.65 (0.03)	1.47	6.85 (0.51)
**7 sat.**	**3i**	2.00 (0.15)	0.92	1.93 (0.06)	0.96	1.90 (0.01)	0.97	1.98 (0.09)	0.93	1.85 (0.16)
**8 sat.**	**3j**	2.77 (0.04)	**2.31**	2.81 (0.11)	**2.28**	2.72 (0.09)	**2.32**	2.76 (0.02)	**2.32**	6.40 (0.03)
**9 sat.**	**3k**	2.61 (0.07)	0.74	2.66 (0.01)	0.73	2.45 (0.01)	0.79	2.10 (0.04)	0.92	1.94 (0.07)
**10 sat.**	**3l**	5.91 (0.02)	0.61	5.95 (0.04)	0.61	5.95 (0.02)	0.61	5.47 (0.08)	0.66	3.62 (0.04)
**11 sat.**	**3m**	1.78 (0.02)	**2.59**	2.31 (0.13)	**2.00**	1.76 (0.01)	**2.62**	1.83 (0.08)	**2.52**	4.62 (0.04)
**12 sat.**	**3n**	9.69 (0.14)	1.52	10.24 (0.12)	1.43	9.49 (0.11)	1.55	10.15 (0.27)	1.45	14.70 (0.09)

**Legend**: **OA**—oleanolic acid (reference compound); **sat.**—saturated linker; **unsat.**—unsaturated linker; **IC_50_**—half maximal inhibitory concentration; **SD**—the standard deviation; **SKBR-3**—human breast adenocarcinoma; **SKOV-3**—human ovarian cystadenocarcinoma); **PC-3**—human prostate carcinoma; **U-87**—human glioblastoma.

#### 2.3.2. Selectivity Index

The results of the Selectivity Index, calculated as IC_50_ for normal cell line (HDF)/IC_50_ for respective cancerous cell line, are given in Table 3.

### 2.4. Antioxidant Activity of Acetylated Oleanolic Acid Dimers (AcOADs) ***3a**–**3n***

**Figure 3:**
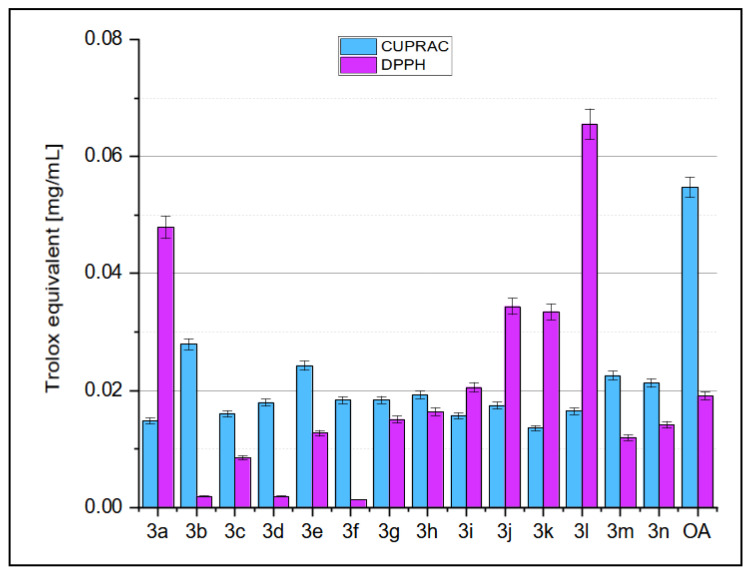
The ability to inhibit the DPPH and CUPRAC radicals by AcOADs **3a**–**3n** and oleanolic acid (**1**, OA) expressed as Trolox equivalent.

The results of the antioxidant activity of oleanolic acid (**1**) and Acetylated Oleanolic Acid Dimers (AcOADs) **3a**–**3n** evaluated with CUPRAC and DPPH assays [42,46] are given in Figure 3. The results are presented as % inhibition of the copper(II) ions and Trolox equivalent (blue bars) calculated from the standard curve, presented in the Supplementary Materials (Supplementary Materials S02, Figure S1) and as % inhibition of the DPPH radical and Trolox equivalent (pink bars), calculated from the standard curve presented in the Supplementary Materials (Supplementary Materials S02, Figure S2).

## 3. Discussion

### 3.1. Synthesis of Acetylated Oleanolic Acid Dimers (AcOADs) ***3a**–**3n***

#### 3.1.1. Acetylated Oleanolic Acid Dimers (AcOADs) **3a**–**3n** Preparation

Acetylation of oleanolic acid derivatives is generally carried out in pyridine at room temperature, e.g., [47]; however, in our work, the synthesis procedure was modified to avoid the use of toxic pyridine. Two methods of modification were performed: (i) a saturated solution of triterpene dimer in acetic anhydride was heated for 15 min, then immediately filtered (without cooling) and allowed to crystallize; (ii) a saturated solution of triterpene dimer in acetic anhydride was heated for 15 min, then poured into 5-volume of water, filtered off, washed with water and dried. Crude products were crystallized from ethanol. If crystallization failed, the product was precipitated with water from an ethanol solution.

#### 3.1.2. The Influence of Linker Structure of AcOADs **3a**–**3n** on Their Melting Point

All Acetylated Oleanolic Acid Dimers (AcOADs) were crystallized from ethanol after being isolated from the mixture after the reaction; in case of failure—they were precipitated with water from an ethanol solution.

**Figure 4:**
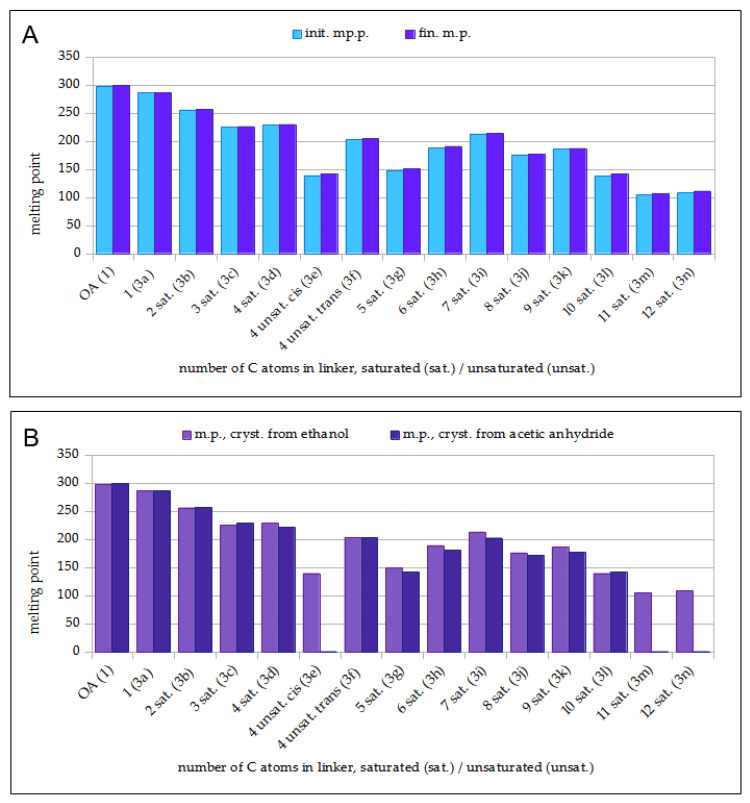
The dependency between the melting point of AcOADs (**3a**–**3n**) and the number of carbon atoms in the linker joining two triterpene moieties: (**A**) AcOADs crystallized/precipitated from ethanol; (**B**) AcOADs crystallized from ethanol (light violet bars) and acetic anhydride (dark violet bars). **Legend**: **OA**—oleanolic acid (reference compound); **sat.**—saturated linker; **unsat.**—unsaturated linker; **init. m.p.**—initial melting point; **fin. m.p.**—final melting point; **m.p., cryst. from ethanol**—melting point of dimer crystallized from ethanol; **m.p., cryst. from acetic anhydride**—the melting point of dimer crystallized from acetic anhydride.

Figure 4A shows the dependence between the length of the linker connecting two acetyloleanolic acid moieties (X-axis) and the melting point of the crystallized/precipitated AcOADs (Y axis).

All AcOADs with short and moderately long linkers (**3a**–**3l**, Figure 2) crystallized from ethanol, and it was observed that as the length of the chain joining two triterpene units increased, the solubility of the dimer in ethanol also increased, while the susceptibility to crystallization decreased. Two AcOADs with the longest linkers, with eleven- and twelve-carbon atoms (compounds **3m** and **3n**, respectively), were well soluble in ethanol even at room temperature and did not crystallize, so these compounds were precipitated with water from an ethanol solution.

The melting points of all AcOADs (**3a**–**3n**) were lower than the melting point of oleanolic acid (298–300 °C) and were in the range of 108–287 °C. Considering the AcOADs with saturated linkers (**3a**–**3e**, **3g**–**3n**), it is quite clear that with an increase in the number of carbon atoms in the linker connecting two acetyloleanolic acid residues (dimers **3a**–**3n**), the melting point decreases; AcOADs **3e** (with a *cis*-unsaturated four-carbon linker), **5g** (with a five-carbon linker) and **5i** (dimer with a seven-carbon linker) break out from the above rule.

For *cis* and *trans* isomers (compounds **3e**, **3f,** respectively), the *trans* isomer showed a higher melting point than the *cis* isomer, which is consistent with the general rules [48].

Figure 4B shows a comparison of the dependency of the linker’s length of AcOADs (**3a**–**3n**) (X-axis) on the melting point of the dimer crystallized from ethanol (light violet bars) and from acetic anhydride (dark violet bars) (Y axis).

Comparing the melting points of disubstituted dimers (**3a**–**3n**), crystallized from ethanol and crystallized from acetic anhydride (Figure 4B), it can be easily seen that in most cases, these temperatures are very similar. The exceptions are dimers **3e** (with a *cis*-unsaturated linker), **3m,** and **3n** (with an 11- and 12-carbon linker, respectively), which did not crystallize from acetic anhydride.

**Figure 5:**
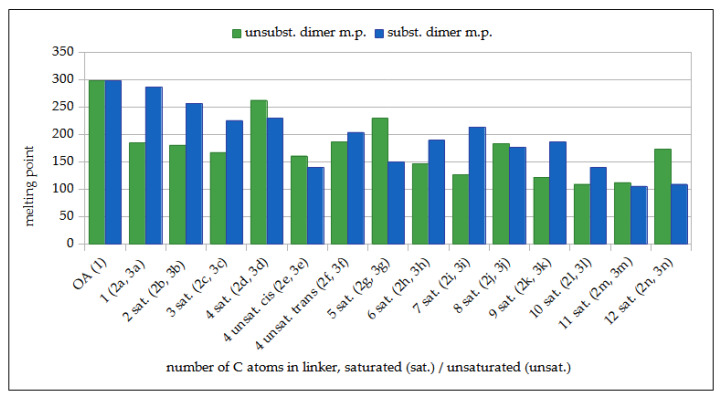
The comparison of the dependency between the melting point of OADs **2a**–**2n** (green bars) [41] and AcOADs **3a**–**3n** (blue bars) and the length of carbon atoms in the linker joining two triterpene moieties. **Legend**: **OA**—oleanolic acid (reference compound); **sat.**—saturated linker; **unsat.**—unsaturated linker; **unsubst. dimer m.p.**—melting point of the unsubstituted dimer; **subst. dimer m.p.**—melting point of the disubstituted dimer.

Figure 5 shows a comparison of the dependency between the length of the linker connecting two oleanolic acid moieties (OADs **2a**–**2n**, green bars, [41]) and acetyloleanolic acid moieties (AcOADs **3a**–**3n**, blue bars) (X-axis) and the melting point of the purified dimer of both series (Y axis).

For AcOADs **3a**–**3c**, **3f**, **3h**, **3i**, **3k,** and **3l**, the introduction of two CH_3_CO- groups into the dimer molecule resulted in an increase in the melting point of the product, while in the remaining cases, the acetylation product showed a lower melting point than the corresponding substrates.

#### 3.1.3. The Polarity of Acetylated Oleanolic Acid Dimers (AcOADs) **3a**–**3n**

The polarity of AcOADs **3a**–**3n** was clearly less polar than the polarity of oleanolic (**1**) and acetyloleanolic acids.

Comparing the R_f_ values of AcOADs **3a**–**3n** (Table 4), it can be observed that the polarity of these compounds was very similar. Only the disubstituted dimer with a one-carbon linker (**3a**) and, to a lesser extent, the two AcOADs with the longest linkers (**3m** and **3n**) were slightly less polar than the other AcOADs (**3b**–**3l**). The spots from the two AcOADs with four-carbon bridges containing the unsaturated bond (**3e** and **3f**) had almost the same R_f_ as the spots of most of the other AcOADs, so the polarity of these compounds is very similar.

#### 3.1.4. Spectral Characterization of Acetylated Oleanolic Acid Dimers (AcOADs) **3a**–**3n**

Spectral Characterization of Disubstituted Dimer **3a** (with One-Carbon Linker)

Graphic forms of the ^1^H NMR, ^13^C NMR, and DEPT spectra recorded for AcOAD **3a**, which is representative of the “3” series of dimers, are provided in Supplementary Materials (Supplementary Materials S03).

Summary of IR Spectral Data of Acetylated Oleanolic Acid Dimers (AcOADs) **3a**–**3n**

In all IR spectra of AcOADs **3a**–**3n**, compared to the IR spectra of unsubstituted dimers **2a**–**2n** [41], two additional absorption bands were observed, indicating the presence of the CH_3_COO- group instead of the -OH group at the C-3 position. The first of them was observed at ν 1717–1720 cm^−1^ and came from the carbonyl group within the acetoxy groups (CH_3_COO-), while the second one was present at ν 1453–1456 cm^−1^ and came from the stretching vibrations of the C-O- group at the CH_3_COO- function (Table 5).

Summary of 1H NMR Spectral Data of Acetylated Oleanolic Acid Dimers (AcOADs) **3a**–**3n**

In the ^1^H NMR spectra of AcOADs (**3a**–**3n**), the presence of all signals characteristic for the oleanane skeleton was found (Table 6), with the signal assigned to the proton in the C-3 position being at a slightly higher chemical shift value than in the molecule of, e.g., oleanolic acid (**1**). Moreover, the strong singlet derived from the CH_3_ group within CH_3_COO- moiety was observed in the ^1^H NMR spectra of dimers **3a**–**3n** at δ 2.03–2.06 ppm (Table 6).

The signal derived from protons attached to the extreme carbon atoms within the linkers of AcOADs **3b–3n** was usually observed in the range of δ 4.00–4.63 ppm (Table 6), and only for the dimer with a one-carbon linker (**3a**) at δ 5.73 ppm. This signal was most often observed as a multiplet (for dimers: **3b**, **3d**, **3i**, **3j**, **3k**, **3l,** and **3n**) or a triplet (for dimers: **3c**, **3f**, **3g,** and **3h**), rarely as a triplet of doublets (for dimer **3m**). Only for the dimer with the shortest linker (**3a**) the discussed signal was observed in the form of a singlet, while for the dimer **3e**, with a four-carbon linker containing a *cis*-unsaturated bond, the discussed signal was present in the form of a doublet of doublets of doublets (ddd).

Summary of 13C NMR Spectral Data of Acetylated Oleanolic Acid Dimers (AcOADs) **3a**–**3n**

The values of chemical shifts for the most important signals present in the ^13^C NMR spectra of AcOADs **3a**–**3n**, characteristic for the oleanane system and the extreme atoms of the linker connecting two triterpene residues, are presented in Table 7. For comparison, the relevant spectral data for oleanolic acid (**1**) are also given [49].

In the ^13^C NMR spectra of AcOADs (**3a**–**3n**), signals originating from the characteristic carbon atoms of the oleanane system were observed at typical values of chemical shifts [49], namely: δ ~122 ppm (C-12), δ ~144 ppm (C-13) and δ ~46 ppm (C-17) (Table 7). The signal derived from the C-3 carbon atom was observed at the value of chemical shift characteristic for acetyl derivatives, e.g., [44], i.e., at δ of about 81 ppm. In addition, in the analyzed ^13^C NMR spectra of dimers **3a**–**3n**, the presence of two additional signals was found at δ of about 171 and 21 ppm. These are chemical shift values typical for the CH_3_COO- group [44].

The extreme carbon atoms in the linkers of AcOADs **3a**–**3n** formed a signal observed in the range of δ 60–64 ppm, except for the derivative with a bridge containing an unsaturated bond in the *cis* system (**3e**), for which the signal was observed with a significantly lower chemical shift value (δ 59.60 ppm), and also with the exception of the derivative with a one-carbon bridge (**3a**, δ 79.36 ppm).

### 3.2. SAR Analysis of Acetylated Oleanolic Acid Dimers (AcOADs) ***3a**–**3n***

The Structure–Activity Relationship Analysis was performed with the application of a PASS (prediction of activity spectra for substance) computer system [45]. The web-based application predicts the biological activity spectrum of a compound based on its structure. It works on the principle that the biological activity of a compound equates to its structure. The activity of the molecule is predicted by “comparing” the structure of the new compound with the structure of a well-known biological active substrate existing in the database [50].

The probability of occurrence of a given activity is defined as P_a_, and the probability of non-occurrence of this activity is defined as P_i_. Both values are expressed in a range between 0 and 1. If, for a given compound, the value of P_a_ is greater than 0.700, it indicates a very high probability that this compound will also show this activity in experimental studies and will prove to be a functional analog of an existing drug. If the P_a_ value is contained in the range of 0.500–0.700, there is a significant probability that the substance will show such activity experimentally but will not be structurally similar to known active substances. Where the calculated probability occurrence of a given activity will be below 0.500, there is only a small chance that the analyzed compound will show a certain biological activity under the conditions of the experiment, but if the experiment confirms such action, the given compound may become a New Leading Structure [50].

The negative control sample in our computational studies was the parent compound, i.e., oleanolic acid (**1**). This statement is based on many scientific publications in which various directions of pharmacological activity of oleanolic acid (**1**) and its derivatives were compared. Of course, it happened (but rarely) that the parent compound (i.e., oleanolic acid, **1**) was slightly more active than the derivatives obtained from it, but most often, the derivatives were much more active compounds. Excellent support for this thesis is, for example, our publications in which we presented the chemical transformations of oleanolic acid and research on the pharmacological activity, mainly anticancer, of these derivatives and the parent compound (e.g., [41,42,44].

Table 1 and Table 2 show the results of predicted biological activities with P_a_ ≥ 0.900 for oleanolic acid (**1**) and AcOADs (compounds **3a**–**3n**). The results of predicted biological activities with P_a_ ≥ 0.700 for oleanolic acid (**1**) and dimers **3a**–**3n** are presented in the Supplementary data (Supplementary Materials S01, Table S1).

A level of probability of occurrence of a given activity (P_a_) above 0.700 was noted for all 14 AcOADs **3a**–**3n.** For these compounds, the program predicted 33 types of activity, with antineoplastic (against colon cancer and colorectal cancer) and vasodilator peripheral activity shown only by dimer **3a** (with the shortest one-carbon bridge). For 8 types of pharmacological activity (caspase 3 stimulator, hepatoprotectant, insulin promoter, lipid metabolism regulator, membrane integrity antagonist, oxidoreductase inhibitor, transcriptor factor NF kappa B stimulator, and transcriptor factor stimulator), the level of activity above 0.900 was predicted for almost all 14 AcOADs (**3a**–**3n**). For some predicted activities, AcOADs with an unsaturated linker (**3e** and **3f**) behaved atypically—as the only, or almost the only, out of 14 AcOADs, they showed lower activity than the other dimers (anti-inflammatory, antipruritic, antitussive, lipid peroxidase inhibitor), or clearly higher (anti-ulcerative, chemopreventive, cytoprotectant). It was observed that only for some types of biological activity and only for dimers containing from one to three or four -CH_2_- groups in the linker, as the linker lengthens, there is a clear increase or decrease in the level of biological activity.

Comparing the results of the predicted biological activities for unsubstituted dimers of oleanolic acid (**2a**–**2n**, [41]) and their acetyl derivatives (**3a**–**3n**), it can be seen that the introduction of acetyl groups to the dimer molecule causes the following effects:

- the appearance of new types of biological activity, e.g., antineoplastic (against colon and colorectal cancers), cholesterol antagonist;
- loss of activity, e.g., diacylglycerol O-acyl transferase inhibitor, phospholipase inhibitors;
- does not significantly affect the level of some activities, e.g., acylcarnityne hydrolase inhibitors, anti-inflammatory, antipruritic, chemopreventive;
- reduction of the level of some activities, e.g., antitussive, chitinase inhibitors, cytoprotective;
- increasing the level of certain activities, e.g., antisecretoric, mucomebranous protector.

In summary, AcOADs **3a**–**3n** may exhibit several valuable pharmacological activities in vitro, with the highest levels of these activities expected from short-linker dimers containing four or fewer saturated carbon atoms.

### 3.3. Cytotoxic Activity of Acetylated Oleanolic Acid Dimers (AcOADs) ***3a**–**3n***

#### 3.3.1. MTT Assay

Comparing the IC_50_ values obtained for four cancer cell lines SKRB-3, SKOV-3, PC-3, and U-87, which were treated with OADs (**2a**–**2n**, [41]) and AcOADs (**3a**–**3n**, Figure 2), it can be observed that the introduction to a dimer molecule of two acetyl residues causes an increase in the level of cytotoxic activity in the case of most dimers (Table 3). The least active turned out to be AcOADs with the longest twelve-carbon chain. The dimer with a four-carbon *trans*-unsaturated bridge **3f** and dimers with a six- and ten-carbon linker (**3h** and **3l**, respectively) were approximately 3 times more active than oleanolic acid (**1**), and the IC_50_ value for these three compounds ranged from 4.65 to 7.06 μM. Six AcOADs, i.e., **3b** (with a two-carbon linker), **3c** (with a three-carbon linker), **3d** (with a saturated four-carbon linker), **3e** (with a four-carbon *cis*-unsaturated linker), **3g** (with a five-carbon linker) and **3j** (with an eight-carbon linker) showed an IC_50_ value of 2.5–4.5 μM, which means that the level of cytotoxic activity towards the four cancer cell lines tested was 4 to 6.5 times higher than that of oleanolic acid (**1**). The highest activity, from 7 to over 9 times higher than oleanolic acid (**1**), was demonstrated by four AcOADs, i.e., **3a** (with a one-carbon linker), **3i** (with a seven-carbon linker), **3k** (with a nine-carbon linker) and **3m** (with an eleven-carbon linker). The IC_50_ value for the four dimers mentioned (**3a**, **3i**, **3k,** and **3m**) was around 2 μM. Particularly noteworthy are dimers **3i** and **3m**, for which, in tests on almost all four cell lines, the IC_50_ value did not exceed 2 μM (Table 3).

#### 3.3.2. Selectivity Index

Table 3 presents values of the Selectivity Index (SI) for oleanolic acid (**1**) and AcOADs **3a**–**3n**, expressed as a simple ratio of IC_50_ calculated for healthy and cancer cells [51].

The SI value is a very valuable indication in studies mainly concerning cytotoxic/anticancer activity of both preparations from raw materials of natural origin, single chemical substances isolated from such raw materials, and chemically modified compounds of natural origin. In some cases, the SI value is even a deciding factor in whether research on a medicinal preparation or chemical substance will be carried out further. Pena-Moran et al. believe that the limit value of the Selectivity Index determining the validity of further research is at least 10 [52]. In turn, Valderrama and co-workers stated that for individual substances that would become potential anticancer agents, the Selectivity Index limit value is at least 2 [53].

Of the 14 AcOADs tested (**3a**–**3n**), as many as 10 dimers showed a Selectivity Index value higher than 1.00 (**3a**, **3b**, **3d**, **3e**, **3g**, **3h**, **3j**, **3m,** and **2n**) and the next two (**3c** and **3i**) showed an SI value slightly lower than 1 (Table 3). According to Valderrama et al. [53], as many as five diacetylated oleanolic acid dimers, i.e., **3a**, **3b**, **3e**, **3j,** and **3m** (with a one-, two-, eight- or eleven-carbon linker, respectively), have a chance to become potential anticancer compounds. For the AcOADs mentioned, the Selectivity Index value exceeded 2, and for the **3a** dimer, with the shortest, one-carbon bridge, it was approximately 3 (Table 3).

Comparing the SI value for dimers with a four-carbon unsaturated linker (*cis* and *trans*, **3e** and **3f**, respectively), it was found that the *cis* dimer (**3e**) is a highly selective anticancer agent (SI about 2), while the *trans* isomer (**3f**) showed a weak selectivity towards all four cancer cell lines, the SI value for the dimer **3f** was only about 0.5 (Table 3).

### 3.4. Antioxidant Activity of Acetylated Oleanolic Acid Dimers (AcOADs) ***3a**–**3n***

Evaluating antioxidant activity using the DPPH and CUPRAC assays provides complementary insights into the radical scavenging capabilities of a new set of synthesized oleanolic acid derivatives. These assays reveal distinct differences in the antioxidant potential of the compounds when compared to each other and the natural compound, oleanolic acid (OA). In the CUPRAC assay, OA displayed a significantly higher antioxidant activity (0.05474 ± 0.00171 Trolox equivalents) compared to the DPPH assay (0.01907 ± 0.000650639 Trolox equivalents), indicating an overall solid reducing power but a moderate ability to donate hydrogen atoms to DPPH radicals (Figure 3). Among the synthesized derivatives, compounds **3b**, **3e**, and **3m** showed the highest CUPRAC activity, suggesting significant reducing capacity, although they did not surpass the activity of OA. Conversely, in the DPPH assay, derivatives **3a**, **3l**, **3j**, and **3k** exhibited higher antioxidant activity than OA, with **3l** demonstrating the highest activity, indicating their effectiveness at donating hydrogen atoms to neutralize DPPH radicals (Figure 3).

The distinct results between the CUPRAC and DPPH assays highlight the varied antioxidant mechanisms of these compounds. For instance, compound **3a** demonstrated high DPPH activity but only moderate CUPRAC activity, while **3b** showed high CUPRAC activity but low DPPH activity (Figure 3). This variation suggests that dimer **3a** is particularly effective at hydrogen donation, whereas dimer **3b** possesses a strong overall reducing power due to different electron-donating capabilities. This pattern of differential activity is also seen in derivatives **3e** and **3l**, which show relatively balanced activities across both assays, although **3l** exhibited extremely high DPPH activity, suggesting a specialized hydrogen donation mechanism.

Compared to previously analyzed derivatives marked by the number **2** [41,42], these new derivatives (marked by the number **3**) exhibit a broader range of antioxidant activities. In the previous analysis, derivatives **2g**, **2i**, **2j**, and **2m** showed high CUPRAC values, similar to **3b** and **3e**, indicating a robust reducing capacity. However, derivatives like **2a**, which showed high DPPH activity but lower CUPRAC activity [41,42], parallel the behavior of derivative **3a** in the current set. This comparison underscores the importance of structural modifications in influencing antioxidant activity and highlights the need for a comprehensive approach to evaluating these activities using multiple assays.

In conclusion, while oleanolic acid remains a potent antioxidant, certain synthesized derivatives from the current set (marked by **3**) exhibit superior activity in specific assays compared to previously analyzed derivatives (marked by **2**) [41,42]. The variability in performance across the CUPRAC and DPPH assays emphasizes the diverse antioxidant mechanisms and the necessity of a multifaceted approach to evaluating antioxidant capacity. Future research should focus on understanding the structural features that drive these differences and exploring the potential therapeutic applications of the most promising derivatives.

### 3.5. Potentials and Limitations Concerning Acetylated Oleanolic Acid Dimers (AcOADs) ***3a**–**3n***

The obtained oleanolic acid dimers (OADs) and their acyl derivatives (AcOADs) show high potential for future studies in clinical trials. This potential is expressed in the following ways:

1. **Exceptional cytotoxic efficacy**: Our studies have shown that acetylated oleanolic acid dimers (AcOADs) exhibit potent cytotoxic activity against various cancer cell lines, including SKBR-3, SKOV-3, PC-3, and U-87. Many of the tested compounds had IC_50_ values below 5.00 µM, suggesting their high effectiveness. It is important to note that such efficacy in in vitro studies is often a preliminary indicator of potential therapeutic value in in vivo studies.
2. **High selectivity of action**: It is worth emphasizing that some of the tested compounds also showed a favorable Selectivity Index (SI), which means that they are more toxic to cancer cells than to healthy cells. This action profile is desirable in the context of the development of new anticancer drugs, as it minimizes the risk of damage to healthy tissues during therapy.
3. **Antioxidant activity**: In addition to cytotoxic activity, AcOADs have also shown significant antioxidant potential, which may further enhance their therapeutic value. It is known that oxidative stress plays an important role in the pathogenesis of many diseases, including cancer. Dual-acting compounds—cytotoxic and antioxidant—may offer an advantage in cancer therapy by helping to reduce free radical damage and improve the overall condition of patients.
4. **Possibility of structural optimization**: Structure–activity studies (SAR) indicate the possibility of further optimization of these compounds, which may lead to an increase in their pharmacological activity and improvement of pharmacokinetic properties. Such optimization could increase their bioavailability and stability and reduce potential side effects, which is crucial in the context of drug development.
5. **Future preclinical and clinical studies**: Based on the above results, we believe that AcOADs have great potential to advance to in vivo preclinical studies that will allow for the evaluation of their performance in animal models, safety, toxicity, and mechanisms of action. Following positive results from preclinical studies, consideration could be given to start initial clinical trials (Phase I) in small patient groups to assess safety, tolerability, and preliminary efficacy.
6. **Potential Therapeutic Applications**: Due to their broad spectrum of anticancer activity and antioxidant properties, AcOADs may be considered candidates for the therapy of various types of cancers, including those that are difficult to treat, such as those resistant to standard therapies. Moreover, their potential use in combination therapy with other anticancer drugs may open new directions in clinical research.

In summary, AcOADs have significant potential as promising new therapeutic agents for cancer treatment. Further research should focus on optimizing their structure, assessing safety in animal models, and, in the future, on clinical trials, which will allow for a full understanding of their therapeutic potential.

Despite this extremely promising potential, some limitations may be noticed. They concern the following aspects:

1. **Limited number of compounds**: In our study, we focused on the synthesis and characterization of only a dozen or so dimeric oleanolic acid derivatives in which only one type of chemical transformation was performed at the C-3 position, which limits the possibility of fully understanding the impact of various chemical modifications on their biological activity. Although the obtained results are promising, it is necessary to extend the research to a larger number of compounds resulting from other chemical modifications, not only at the C-3 position but, e.g., at the C-11 or C-12 position, to better understand the relationship between structure and activity.
2. **In vitro tests**: All experiments regarding anticancer and antioxidant activity were performed on cell lines in vitro. The results of these studies, although promising, do not always translate directly into effectiveness in living organisms. Future studies should include in vivo studies to assess the bioavailability, toxicity, and therapeutic effectiveness of these compounds in animal models.
3. **Lack of analysis of mechanisms of action**: In the presented work, we focused mainly on the assessment of biological activity and physicochemical characteristics of new compounds. However, we have not conducted detailed studies of the mechanisms of pharmacological action that would allow us to understand the precise molecular pathways through which these compounds exert their anticancer and antioxidant effects. In future studies, we plan to analyze the mechanisms of action, which will help to better understand the therapeutic potential of these compounds.
4. **Lack of long-term stability studies**: Long-term stability studies of the new derivatives were not carried out in this study. The stability of chemical compounds is crucial for their potential clinical use. We intend to address this aspect in future work, which will allow us to better determine the suitability of these compounds as drug candidates.
5. **Poor water solubility**: One of the main challenges in developing new oleanolic dimeric acid (OAD) derivatives is their limited water solubility. Although this aspect may affect the bioavailability of compounds in vivo, it is worth emphasizing that most potential drugs face similar challenges in the early stages of research. Our study provides important information on the structure and activity of these compounds, which can provide a solid basis for further structural modifications. These modifications have the potential to improve water solubility and thus increase their usefulness in clinical applications. We are also considering conducting research that will enable us to solve the problem of the lack of solubility of triterpene derivatives in water, e.g., with the application of liposomes, nanocomplexes, or other techniques.

In conclusion, although there are some limitations, such as poor water solubility, our study provides valuable information and provides a solid basis for future work on the improvement of these compounds. We believe that further research will help overcome these challenges and contribute to the development of effective anticancer drugs.

Considering the above limitations will help a better understanding of the results and design future studies that could overcome these challenges.

## 4. Materials and Methods

### 4.1. Materials

#### 4.1.1. NMR

The ^1^H- and ^13^C-NMR spectra were recorded using a Bruker Advance 600 MHz spectrometer (Billerica, MA, USA). Chemical shifts (δ) were expressed in parts per million (ppm) relative to tetramethylsilane (TMS) as an internal standard, using CDCl_3_ as a solvent. Coupling constants (*J*) are expressed in Hertz (Hz).

#### 4.1.2. Syntheses, TLC and HP TLC and Antioxidant Activity

All commercially available solvents and reagents used in our experiments were graded “pure for analysis” (Aldrich^®^, Darmstadt Germany; Fluka^®^, Charlotte, NC, USA; Chempur^®^, Piekary Śląskie, Poland; and POCh^®^, Gliwice, Poland). The solvents were dried according to the usual procedures. Oleanolic Acid Dimers (OADs) were obtained according to the earlier described procedure [41].

#### 4.1.3. MTT Assay

Human cancer cells: SKBR-3 and SKOV-3 were cultured in McCoy’s Modified Medium; PC-3 was cultured in an F-12K medium; U-87 was cultured in EMEM medium; HDF was cultured in Fibroblast Basal Medium. Each medium was supplemented with 10% fetal bovine serum, 1% L- glutamine, and 1% penicillin/streptomycin solution. The cell lines were kept in an incubator at 37 °C. All the cell lines and mediums were obtained from the American Type Culture Collection (ATCC) supplied by LGC-Standards (Lomianki, Poland).

#### 4.1.4. Antioxidant Activity

The CUPRAC reagent (7.5 mM ethanolic 96% neocuproine solution, 10 mM CuCl_2 ×_ H_2_O solution, and an ammonium acetate buffer of pH 7.0) and DPPH reagent (2,2-diphenyl-1-picrylhydrazyl 0.2 mM solution) were applied.

### 4.2. Methods

#### 4.2.1. Preparation of AcOADs—General Method A

A saturated solution of dimers **2a**–**2n** [41] in hot acetic anhydride was refluxed until total consumption of starting material (TLC control; usually 15 min). The obtained hot solution was filtered and left for crystallization or precipitated with water, filtered off, and dried.

#### 4.2.2. Preparation of AcOADs—General Method B

A saturated solution of dimers **2a**–**2n** [41] in hot acetic anhydride was refluxed until total consumption of starting material (TLC control; usually 15 min). The obtained hot solution was poured into a 5-time volume of water. The resulting precipitate was filtered off, washed with water, dried, and crystallized from ethanol or re-precipitated with water from ethanolic solution.

Spectral characteristics presented below contain only the signals most characteristic for the molecules of the obtained compounds **3a**–**3n**.

**Dimer 3a**: C_65_H_100_O_8_; mol. mass: 1008.74; yields: 960 mg (=94.9%); m.p.: 286–288 °C (Met. 1), 287–290. °C (Met. 2, EtOH); **IR** (ν, cm^−1^): 1730.55 and 1718.66 (2 × C=O), 1461.76 and 1454.02 (2 × C-O-). **^1^H NMR** (δ, ppm): 5.73 (2H, s, -O-CH_2_-O-), 5.28 (2H, t, *J* = 3.4 Hz, 2 × C_12_-H), 4.48 (2H, dd, *J* = 9.4 and 4.8 Hz, 2 × C_3_-H_α_), 2.82 (2H, dd, *J* = 13.5 and 13.5 Hz, 2 × C_18_-H_β_), 2.03 (6H, s, 2 × CH_3_COO-), 1.12, 0.92, 0.90, 0.89, 0.86, 0.85, 0.74 (7 × 6H, 7 × s, 14 CH_3_ groups). **^13^C NMR** (δ, ppm): 176.31 (2 × C_q_, 2 × C-28), 171.03 (2 × C_q_, 2 × CH_3_COO-), 143.35 (2 × C_q_, 2 × C-13), 122.54 (2 × CH, 2 × C-12), 80.90 (2 × CH, 2 × C-3), 79.36 (1 × CH_2_, -O-CH_2_-O-), 46.75 (2 × C_q_, 2 × C-17), 21.29 (2 × CH_3_, 2 × CH_3_COO-). **DEPT**: CH_3_: 8 × 2 CH_3_ (8 signals, 16 CH_3_ groups), CH_2_: 10 × 2 CH_2_ + 1 × 1 CH_2_ (11 signals, 21 CH_2_ groups), CH: 5 × 2 CH (5 signals, 10 CH groups).

**Dimer 3b**: C_66_H_102_O_8_; mol. mass: 1022.76 yields: 930 mg (= 90.7%); m.p.: 257–258 °C (Met. 1), 256–256.5 °C (Met. 2, EtOH); **IR** (ν, cm^−1^): 1730.49 and 1718.46 (2 × C=O), 1461.95 and 1453.93 (2 × C-O-); **^1^H NMR** (δ, ppm): 5.29 (2H, t, *J* = 3.2 Hz, 2 × C_12_-H), 4.49 (2H, dd, *J* = 8.4 and 7.2 Hz, 2 × C_3_-H_α_), 4.30–4.08 (4H, m, -O-CH_2_-CH_2_-O-), 2.86 (2H, dd, *J* = 13.2 and 3.7 Hz, 2 × C_18_-H_β_), 2.05 (6H, s, 2 × CH_3_COO-), 1.13, 0.93, 0.92, 0.91, 0.87, 0.85, 0.73 (7 × 6H, 7 × s, 14 CH_3_ groups); **^13^C NMR** (δ, ppm): 177.40 (2 × C_q_, 2 × C-28), 170.94 (2 × C_q_, 2 × CH_3_COO-), 143.54 (2 × C_q_, 2 × C-13), 122.38 (2 × CH, 2 × C-12), 80.83 (2 × CH, 2 × C-3), 62.14 (2 × CH_2_, -O-CH_2_-CH_2_-O-), 46.66 (2 × C_q_, 2 × C-17), 21.27 (2 × CH_3_, 2 × CH_3_COO-); **DEPT**: CH_3_: 8 × 2 CH_3_ (8 signals, 16 CH_3_ groups), CH_2_: 11 × 2 CH_2_ (11 signals, 22 CH_2_ groups), CH: 5 × 2 CH (5 signals, 10 CH groups).

**Dimer 3c**: C_67_H_104_O_8_; mol. mass: 1036.77; yields: 980 mg (= 94.6%); m.p.: 229–230 °C (Met. 1), 225–226.5 °C (Met. 2, EtOH); **IR** (ν, cm^−1^): 1731.53 and 1718.88 (2 × C=O), 1462.72 and 1454.82 (2 × C-O-); **^1^H NMR** (δ, ppm): 5.28 (2H, t, *J* = 3.3 Hz, 2 × C_12_-H), 4.49 (2H, dd, *J* = 9.7 and 7.9 Hz, 2 × C_3_-H_α_), 4.09 (4H, t, *J* = 6.3 Hz, -O-CH_2_-CH_2_-CH_2_-O-), 2.85 (2H, dd, *J* = 13.6 and 3.9 Hz, C_18_-H_β_), 2.04 (6H, s, 2 × CH_3_COO-), 1.98–1.93 (2H, m, -O-CH_2_-CH_2_-CH_2_-O-), 1.13, 0.93, 0.92, 0.90, 0.86, 0.85, 0.73 (7 × 6H, 7 × s, 14 × CH_3_ groups); **^13^C NMR** (δ, ppm): 177.45 (2 × C_q_, 2 × C-28), 170.92 (2 × C_q_, 2 × CH_3_COO-), 143.63 (2 × C_q_, 2 × C-13), 122.26 (2 × CH, 2 × C-12), 80.78 (2 × CH, 2 × C-3), 60.74 (2 × CH_2_, -O-CH_2_-CH_2_-CH_2_-O-), 46.64 (2 × C_q_, 2 × C-17), 28.05 (1 × CH_2_, -O-CH_2_-CH_2_-CH_2_-O-), 21.25 (2 × CH_3_, 2 × CH_3_COO-); **DEPT**: CH_3_: 8 × 2 CH_3_ (8 signals, 16 CH_3_ groups), CH_2_: 11 × 2 CH_2_ + 1 × 1 CH_2_ (12 signals, 23 CH_2_ groups), CH: 5 × 2 CH (5 signals, 10 CH groups).

**Dimer 3d**: C_68_H_106_O_8_; mol. mass: 1050.79; yields: 978 mg (= 93.1%); m.p.: 221–223 °C (Met. 1), 229–229.5 °C (Met. 2, EtOH); **IR** (ν, cm^−1^): 1730.30 and 1718.02 (2 × C=O), 1462.19 and 1453.54 (2 × C-O-); **^1^H NMR** (δ, ppm): 5.28 (2H, t, *J* = 3.3 Hz, 2 × C_12_-H), 4.49 (2H, dd, *J* = 9.7 and 7.9 Hz, 2 × C_3_-H_α_), 4.09–3.98 (4H, m, -O-CH_2_-CH_2_-CH_2_-CH_2_-O-), 2.86 (2H, dd, *J* = 13.6 and 3.8 Hz, 2 × C_18_-H_β_), 2.05 (6H, s, 2 × CH_3_COO-), 1.91–1.82 (4H, m, -O-CH_2_-CH_2_-CH_2_-CH_2_-O-), 1.13, 0.93, 0.92, 0.90, 0.86, 0.85, 0.73 (7 × 6H, 7 × s, 7 CH_3_ groups); **^13^C NMR** (δ, ppm): 177.59 (2 × C_q_, 2 × C-28), 170.95 (2 × C_q_, 2 × CH_3_COO-), 143.72 (2 × C_q_, 2 × C-13), 122.23 (2 × CH, 2 × C12), 80.80 (2 × CH, 2 × C-3), 63.62 (2 × CH_2_, -O-CH_2_-CH_2_-CH_2_-CH_2_-O-), 46.63 (2 × C_q_, 2 × C-17), 25.40 (2 × CH_2_, -O-CH_2_-CH_2_-CH_2_-CH_2_-O-), 21.28 (2 × CH_3_, CH_3_COO-); **DEPT**: CH_3_: 8 × 2 CH_3_ (8 signals, 16 CH_3_ groups), CH_2_: 12 × 2 CH_2_ (12 signals, 24 CH_2_ groups), CH: 5 × 2 CH (5 signals, 10 CH groups).

**Dimer 3e**: C_68_H_104_O_8_; mol. mass: 1048.77; yields: 1001 mg (= 95.5%); m.p.:—°C (Met. 1; oil), 139–142 °C (Met. 2, EtOH); **IR** (ν, cm^−1^): 1729.25 (2 × C=O), 1463.43 and 1453.40 (2 × C-O-); **^1^H NMR** (δ, ppm): 5.70 (2H, t, *J* = 4.1 Hz, -O-CH_2_-CH=CH-CH_2_-O-), 5.29 (2H, t, *J* = 3.5 Hz, 2 × C_12_-H), 4.63 (4H, ddd, *J* = 17.9, 13.0 and 4.4 Hz, -O-CH_2_-CH=CH-CH_2_-O-), 4.49 (2H, dd, *J* = 9.5 and 7.5, 2 × C_3_-H_α_), 2.85 (2H, dd, *J* = 13.1 and 4.4 Hz, 2 × C_18_-H_β_), 2.06 (6H, s, CH_3_COO-), 1.14, 0.95, 0.94, 0.91, 0.87, 0.85, 0.72 (7 × 6H, 7 × s, 14 × CH_3_ groups); **^13^C NMR** (δ, ppm): 177.28 (2 × C_q_, 2 × C-28), 170.97 (2 × C_q_, 2 × CH_3_COO-), 143.60 (2 × C_q_, 2 × C-13), 128.21 (2 × CH, -O-CH_2_-CH=CH-CH_2_-O-), 122.29 (2 × CH, 2 × C-12), 80.84 (2 × CH, 2 × C-3), 59.60 (2 × CH_2_, -O-CH_2_-CH=CH-CH_2_-O-), 46.60 (2 × C_q_, 2 × C-17), 21.27 (2 × CH_3_, 2 × CH_3_COO-); **DEPT**: CH_3_: 8 × 2 CH_3_ (8 signals, 16 CH_3_ groups), CH_2_: 11 × 2 CH_2_ (11 signals, 22 CH_2_ groups), CH: 6 × 2 CH (6 signals, 12 CH groups).

**Dimer 3f**: C_68_H_104_O_8_; mol. mass: 1048.77; yields: 1012 mg (= 96.5%); m.p.: 203–204 °C (Met. 1), 203–205 °C (Met. 2, EtOH); **IR** (ν, cm^−1^): 1731.30 and 1719.34 (2 × C=O), 1464.58 and 1456.30 (2 × C-O-); **^1^H NMR** (δ, ppm): 5.84 (2H, t, *J* = 2.8 Hz, -O-CH_2_-CH=CH-CH_2_-O-), 5.30 (2H, t, *J* = 3.2 Hz, 2 × C_12_-H), 4.53 (4H, t, *J* = 13.9 Hz, -O-CH_2_-CH=CH-CH_2_-O-), 4.49 (2H, dd, *J* = 9.5 and 7.5 Hz, 2 × C_3_-H_α_) 2.88 (2H, dd, *J* = 13.7 and 3.9 Hz, 2 × C_18_-H_β_), 2.06 (6H, s, 2 × CH_3_COO-), 1.14, 0.94 × 2, 0.91, 0.87, 0.86, 0.73 (5 × 6H + 1 × 12H, 6 × s, 14 × CH_3_ groups); **^13^C NMR** (δ, ppm): 177.30 (2 × C_q_, 2 × C-28), 171.02 (2 × C_q_, 2 × CH_3_COO-), 143.67 (2 × C_q_, 2 × C-13), 127.94 (2 × CH, -O-CH_2_-CH=CH-CH_2_-O-), 122.36 (2 × CH, 2 × C-12), 80.88 (2 × CH, 2 × C-3), 63.70 (2 × CH_2_, -O-CH_2_-CH=CH-CH_2_-O-), 46.75 (2 × C_q_, 2 × C-17), 21.31 (2 × CH_3_, 2 × CH_3_COO-); **DEPT**: CH_3_: 8 × 2 CH_3_ (8 signals, 16 CH_3_ groups), CH_2_: 11 × 2 CH_2_ (11 signals, 22 CH_2_ groups), CH: 6 × 2 CH (6 signals, 12 CH groups).

**Dimer 3g**: C_69_H_108_O_8_; mol. mass: 1064.80; yields: 1035 mg (= 97.2%); m.p.: 142–144 °C (Met. 1), 148–151 °C (Met. 2, EtOH); **IR** (ν, cm^−1^): 1728.96 and 1718.50 (2 × C=O), 1463.13 and 1454.76 (2 × C-O-); **^1^H NMR (**δ, ppm): 5.28 (2H, t, *J* = 3.2 Hz, 2 × C_12_-H), 4.49 (2H, dd, *J* = 9.2 and 7.9 Hz, 2 × C_3_-H_α_), 4.02 (4H, t, *J* = 6.4 Hz, -O-CH_2_-CH_2_-CH_2_-CH_2_-CH_2_-O-), 2.86 (2H, dd, *J* = 13.8 and 4.0 Hz, 2 × C_18_-H_β_), 2.05 (6H, s, 2 × CH_3_COO-), 1.13, 0.93 × 2, 0.90, 0.86, 0.85, 0.73 (5 × 6H + 1 × 12H, 6 × s, 14 × CH_3_ groups); **^13^C NMR** (δ, ppm): 177.64 (2 × C_q_, 2 × C-28), 170.96 (2 × C_q_, 2 × CH_3_COO-), 143.74 (2 × C_q_, 2 × C-13), 122.22 (2 × CH, 2 × C-12), 80.83 (2 × CH, 2 × C-3), 63.93 (2 × CH_2_, -O-CH_2_-CH_2_-CH_2_-CH_2_-CH_2_-O-), 46.61 (2 × C_q_, 2 × C-17), 28.10 (2 × CH_2_, -O-CH_2_-CH_2_-CH_2_-CH_2_-CH_2_-O-), 22.96 (1 × CH_2_, -O-CH_2_-CH_2_-CH_2_-CH_2_-CH_2_-O-), 21.27 (2 × CH_3_, 2 × CH_3_COO-); **DEPT**: CH_3_: 8 × 2 CH_3_ (8 signals, 16 CH_3_ groups), CH_2_: 12 × 2 CH_2_ + 1 × 1 CH_2_ (13 signals, 25 CH_2_ groups), CH: 5 × 2 CH (5 signals, 10 CH groups).

**Dimer 3h**: C_70_H_110_O_8_; mol. mass: 1078.82; yields: 1013 mg (= 93.9%); m.p.: 180–183 °C (Met. 1), 188–191 °C (Met. 2, EtOH); **IR** (ν, cm^−1^): 1730.48 and 1717.67 (2 × C=O), 1463.84 and 1454.52 (2 × C-O-); **^1^H NMR** (δ, ppm): 5.29 (2H, t, *J* = 3.4 Hz, 2 × C_12_-H), 4.50 (2H, dd, *J* = 7.9 and 4.4 Hz, 2 × C_3_-H_α_), 4.02 (4H, t, *J* = 6.5 Hz, -O-CH_2_-CH_2_-CH_2_-CH_2_-CH_2_-CH_2_-O-), 2.87 (2H, dd, *J* = 14.1 and 4.1 Hz, 2 × C_18_-H_β_), 2.06 (6H, s, 2 × CH_3_COO-), 1.13, 0.93 × 2, 0.90, 0.86, 0.85, 0.73 (5 × 6H + 1 × 12H, 6 × s, 14 × CH_3_ groups); **^13^C NMR** (δ, ppm): 177.59 (2 × C_q_, 2 × C-28), 170.97 (2 × C_q_, 2 × CH_3_COO-), 143.80 (2 × C_q_, 2 × C-13), 122.21 (2 × CH, 2 × C-12), 80.86 (2 × CH, 2 × C-3), 64.03 (2 × CH_2_, -O-CH_2_-CH_2_-CH_2_-CH_2_-CH_2_-CH_2_-O-), 46.64 (2 × C_q_, 2 × C-17), 28.57 × 2 (2 × CH_2_, -O-CH_2_-CH_2_-CH_2_-CH_2_-CH_2_-CH_2_-O-), 27.59 × 2 (2 × CH_2_, -O-CH_2_-CH_2_-CH_2_-CH_2_-CH_2_-CH_2_-O-), 21.27 (2 × CH_3_, 2 × CH_3_COO-); **DEPT**: CH_3_: 8 × 2 CH_3_ (8 signals, 16 CH_3_ groups), CH_2_: 13 × 2 CH_2_ (13 signals, 26 CH_2_ groups), CH: 5 × 2 CH (5 signals, 10 CH groups).

**Dimer 3i**: C_71_H_112_O_8_; mol. mass: 1092.83; yields: 1015 mg (= 92.9%); m.p.: 201–205 °C (Met. 1), 213–214 °C (Met. 2, EtOH); **IR** (ν, cm^−1^): 1729.19 and 1718.95 (2 × C=O), 1463.99 and 1453.76 (2 × C-O-); **^1^H NMR** (δ, ppm): 5.28 (2H, t, *J* = 3.4 Hz, 2 × C_12_-H), 4.50 (2H, dd, *J* = 7.9 and 4.5 Hz, 2 × C_3_-H_α_), 4.05–3.97 (4H, m, -O-CH_2_-CH_2_-CH_2_-CH_2_-CH_2_-CH_2_-CH_2_-O-), 2.88 (2H, dd, *J* = 13.8 and 4.0 Hz, 2 × C_18_-H_β_), 2.06 (6H, s, 2 × CH_3_COO-), 1.14, 0.94, 0.93, 0.91, 0.88, 0.86, 0.74 (7 × 6H, 7 × s, 14 CH_3_ groups); **^13^C NMR (**δ, ppm): 177.74 (2 × C_q_, 2 × C-28), 171.02 (2 × C_q_, 2 × CH_3_COO-), 143.83 (2 × C_q_, 2 × C-13), 122.24 (2 × CH, 2 × C-12), 80.90 (2 × CH, 2 × C-3), 64.15 (2 × CH_2_, -O-CH_2_-CH_2_-CH_2_-CH_2_-CH_2_-CH_2_-CH_2_-O-**)**, 46.67 (2 × C_q_, 2 × C-17), 28.85 (1 × CH_2_, -O-CH_2_-CH_2_-CH_2_-CH_2_-CH_2_-CH_2_-CH_2_-O-), 28.56 (2 × CH_2_, -O-CH_2_-CH_2_-CH_2_-CH_2_-CH_2_-CH_2_-CH_2_-O-), 26.05 (2 × CH_2_, -O-CH_2_-CH_2_-CH_2_-CH_2_-CH_2_-CH_2_-CH_2_-O-), 21.31 (2 × CH_3_, 2 × CH_3_COO-); **DEPT**: CH_3_: 8 × 2 CH_3_ (8 signals, 16 CH_3_ groups), CH_2_: 13 × 2 CH_2_ + 1 × 1 CH_2_ (14 signals, 27 CH_2_ groups), CH: 5 × 2 CH (5 signals, 10 CH groups).

**Dimer 3j**: C_72_H_114_O_8_; mol. mass: 1106.85; yields: 1044 mg (= 94.3%); m.p.: 171–174 °C (Met. 1), 176–177 °C (Met. 2, EtOH); **IR** (ν, cm^−1^): 1729.02 and 1718.4417 (2 × C=O), 1464.02 and 1453.33 (2 × C-O-); **^1^H NMR (**δ, ppm): 5.27 (2H, t, *J* = 3.2 Hz, 2 × C_12_-H), 4.50 (2H, dd, *J* = 7.9 and 4.5 Hz, 2 × C_3_-H_α_), 4.07–3.94 (2H, m, -O-CH_2_-CH_2_-CH_2_-CH_2_-CH_2_-CH_2_-CH_2_-CH_2_-O-), 2.86 (2H, dd, *J* = 13.9 and 3.5 Hz, 2 × C_18_-H_β_), 2.04 (6H, s, 2 × CH_3_COO-), 1.13, 0.93, 0.92, 0.90, 0.86, 0.85, 0.73 (7 × 6H, 7 × s, 14 × CH_3_ groups); **^13^C NMR** (δ, ppm): 177.73 (2 × C_q_, 2 × C-28), 171.02 (2 × C_q_, 2 × CH_3_COO-), 143.81 (2 × C_q_, 2 × C-13), 122.22 (2 × CH, 2 × C-12), 80.90 (2 × CH, 2 × C-3), 64.19 (2x CH_2_, -O-CH_2_-CH_2_-CH_2_-CH_2_-CH_2_-CH_2_-CH_2_-CH_2_-O-), 46.65 (2 × C_q_, 2 × C-17), 2 × 29.17 (CH_2_, -O-CH_2_-CH_2_-CH_2_-CH_2_-CH_2_-CH_2_-CH_2_-CH_2_-O-), 28.59 (2 × CH_2_, -O-CH_2_-CH_2_-CH_2_-CH_2_-CH_2_-CH_2_-CH_2_-CH_2_-O-), 23.51 (2 × CH_2_, -O-CH_2_-CH_2_-CH_2_-CH_2_-CH_2_-CH_2_-CH_2_-CH_2_-O-), 21.30 (CH_3_, CH_3_COO-); **DEPT**: CH_3_: 8 × 2 CH_3_ (8 signals, 16 CH_3_ groups), CH_2_: 14 × 2 CH_2_ (14 signals, 28 CH_2_ groups), CH: 5 × 2 CH (5 signals, 10 CH groups).

**Dimer 3k**: C_73_H_116_O_8_; mol. mass: 1120.87; yields: 1079 mg (= 96.3%); m.p.: 176–179 °C (Met. 1), 186–187 °C (Met. 2, EtOH); **IR** (ν, cm^−1^): 1728.66 and 1720.10 (2 × C=O), 1463.55 and 1454.11 (2 × C-O-); **^1^H NMR (**δ, ppm): 5.28 (2H, t, *J* = 3.2 Hz, 2 × C_12_-H), 4.50 (2H, dd, *J* = 7.9 and 4.4 Hz, 2 × C_3_-H_α_), 4.15–3.94 (4H, m, CH_2_, -O-CH_2_-CH_2_-CH_2_-CH_2_-CH_2_-CH_2_-CH_2_-CH_2_-CH_2_-O-), 2.88 (dd, *J* = 13.6 and 3.9 Hz, 2 × C_18_-H_β_), 2.05 (6H s, 2 × CH_3_COO-), 1.14, 0.94, 0.93, 0.91, 0.87, 0.86, 0.74 (7 × 6H, 7 × singlets, 14 × CH_3_ groups); **^13^C NMR** (δ, ppm): 177.74 (2 × C_q_, 2 × C-28), 170.98 (2 × C_q_, 2 × CH_3_COO-), 143.86 (2 × C_q_, 2 × C-13), 122.24 (2 × CH, 2 × C-12), 80.89 (2 × CH, 2 × C-3), 64.21 (2 × CH_2_, -O-CH_2_-CH_2_-CH_2_-CH_2_-CH_2_-CH_2_-CH_2_-CH_2_-CH_2_-O-), 46.66 (2 × C_q_, 2 × C-17), 29.54 (2 × CH_2_, -O-CH_2_-CH_2_-CH_2_-CH_2_-CH_2_-CH_2_-CH_2_-CH_2_-CH_2_-O-), 29.16 (1 × CH_2_, -O-CH_2_-CH_2_-CH_2_-CH_2_-CH_2_-CH_2_-CH_2_-CH_2_-CH_2_-O-), 28.62 (2 × CH_2_, -O-CH_2_-CH_2_-CH_2_-CH_2_-CH_2_-CH_2_-CH_2_-CH_2_-CH_2_-O-), 26.07 (2 × CH_2_, -O-CH_2_-CH_2_-CH_2_-CH_2_-CH_2_-CH_2_-CH_2_-CH_2_-CH_2_-O-), 21.31 (2 × CH_3_, CH_3_COO-); **DEPT**: CH_3_: 8 × 2 CH_3_ (8 signals, 16 CH_3_ groups), CH_2_: 14 × 2 CH_2_ + 1 × 1 CH_2_ (15 signals, 29 CH_2_ groups), CH: 5 × 2 CH (5 signals, 10 CH groups).

**Dimer 3l**: C_74_H_118_O_8_; mol. mass: 1134.88; yields: 1079 mg (= 95.1%); m.p.: 142–143 °C (Met. 1), 140–142 °C (Met. 2, EtOH); **IR** (ν, cm^−1^): 1730.90 and 1717.99 (2 × C=O), 1464.91 and 1453.72 (2 × C-O-); **^1^H NMR (**δ, ppm): 5.25 (2H, t, *J* = 3.4 Hz, 2 × C_12_-H), 4.49 (2H, dd, *J* = 10.2 and Hz, 2 × C_3_-H_α_), 4.08–3.94 (4H, m, (CH_2_, -O-CH_2_-CH_2_-CH_2_-CH_2_-CH_2_-CH_2_-CH_2_-CH_2_-CH_2_-CH_2_-O-), 2.88 (2H, dd, *J* = 14.4 and 3.2 Hz, 2 × C_18_-H_β_), 2.03 (6H, s, 2 × CH_3_COO), 1.13, 0.94, 0.93, 0.90, 0.87, 0.86, 0.74 (7 × 6H, 7 × singlets, 14 × CH_3_ groups); **^13^C NMR** (δ, ppm): 177.73 (2 × C_q_, 2 × C-28), 170.97 (2 × C_q_, 2 × CH_3_COO-), 143.83 (2 × C_q_, 2 × C-13), 122.22 (2 × CH, 2 × C-12), 80.88 (2 × CH, 2 × C-3), 64.21 (2 × CH_2_, -O-CH_2_-CH_2_-CH_2_-CH_2_-CH_2_-CH_2_-CH_2_-CH_2_-CH_2_-CH_2_-O-), 46.65 (2 × C_q_, 2 × C-17), 29.54 (2 × CH_2_, -O-CH_2_-CH_2_-CH_2_-CH_2_-CH_2_-CH_2_-CH_2_-CH_2_-CH_2_-CH_2_-O-), 29.22 (2 × CH_2_, -O-CH_2_-CH_2_-CH_2_-CH_2_-CH_2_-CH_2_-CH_2_-CH_2_-CH_2_-CH_2_-O-), 28.63 (2 × CH_2_, -O-CH_2_-CH_2_-CH_2_-CH_2_-CH_2_-CH_2_-CH_2_-CH_2_-CH_2_-CH_2_-O-), 26.09 (2 × CH_2_, -O-CH_2_-CH_2_-CH_2_-CH_2_-CH_2_-CH_2_-CH_2_-CH_2_-CH_2_-CH_2_-O-), 21.30 (2 × CH_3_, 2 × CH_3_COO-); **DEPT**: CH_3_: 8 × 2 CH_3_ (8 signals, 16 CH_3_ groups), CH_2_: 15 × 2 CH_2_ (15 signals, 30 CH_2_ groups), CH: 5 × 2 CH (5 signals, 10 CH groups).

**Dimer 3m**: C_75_H_120_O_8_; mol. mass: 1148.90; yields: 1047 mg (= 91.2%); m.p.:—°C (Met. 1; oil), 105–107 °C (Met. 2, precip. with H_2_O from EtOH sol.); **IR** (ν, cm^−1^): 1729.09 and 1718.18 (2 × C=O), 1464.55 and 1453.66 (2 × C-O-); **^1^H NMR** (δ, ppm): 5.27 (2H, t, *J* = 3.7 Hz, 2 × C_12_-H), 4.49 (2H, dd, *J* = 10.1 and 8.7 Hz, 2 × C_3_-H_α_), 4.00 (4H, td, *J* = 6.4 and 3.1 Hz, -O-CH_2_-CH_2_-CH_2_-CH_2_-CH_2_-CH_2_-CH_2_-CH_2_-CH_2_-CH_2_-CH_2_-O-), 2.87 (2H, dd, *J* = 13.3 and 4.6 Hz, 2 × C_18_-H_β_), 2.05 (6H, s, 2 × CH_3_COO-), 1.13, 0.93, 0.92, 0.90, 0.86, 0.85, 0.73 (7 × 6H, 7 × s, 14 CH_3_ groups); **^13^C NMR** (δ, ppm): 177.72 (2 × C_q_, 2 × C-28), 170.93 (2 × C_q_, 2 × CH_3_COO-), 143.83 (2 × C_q_, 2 × C-13), 122.21 (2 × CH, 2 × C-12), 80.87 (2 × CH, 2 × C-3), 64.20 (2 × CH_2_, -O-CH_2_-CH_2_-CH_2_-CH_2_-CH_2_-CH_2_-CH_2_-CH_2_-CH_2_-CH_2_-CH_2_-O-), 46.65 (2 × C_q_, 2 × C-17), 29.60 (2 × CH_2_, -O-CH_2_-CH_2_-CH_2_-CH_2_-CH_2_-CH_2_-CH_2_-CH_2_-CH_2_-CH_2_-CH_2_-O-), 29.58 (1 × CH_2_, -O-CH_2_-CH_2_-CH_2_-CH_2_-CH_2_-CH_2_-CH_2_-CH_2_-CH_2_-CH_2_-CH_2_-O-), 29.17 (2 × CH_2_, -O-CH_2_-CH_2_-CH_2_-CH_2_-CH_2_-CH_2_-CH_2_-CH_2_-CH_2_-CH_2_-CH_2_-O-), 28.60 (2 × CH_2_, -O-CH_2_-CH_2_-CH_2_-CH_2_-CH_2_-CH_2_-CH_2_-CH_2_-CH_2_-CH_2_-CH_2_-O-), 26.05 (2 × CH_2_, -O-CH_2_-CH_2_-CH_2_-CH_2_-CH_2_-CH_2_-CH_2_-CH_2_-CH_2_-CH_2_-CH_2_-O-), 21.30 (2 × CH_3_, 2 × CH_3_COO-); **DEPT**: CH_3_: 8 × 2 CH_3_ (8 signals, 16 CH_3_ groups); CH_2_: 15 × 2 CH_2_ + 1 × 1 CH_2_ (16 signals, 31 CH_2_ groups); CH: 5 × 2 CH (5 signals, 10 CH groups).

**Dimer 3n**: C_76_H_122_O_8_; mol. mass: 1162.91; yields: 1098 mg (= 94.5%); m.p.:—°C (Met. 1; oil), 105–108 °C (Met. 2, precip. with H_2_O from EtOH sol.). **IR** (ν, cm^−1^): 1729.16 and 1718.24 (2 × C=O), 1463.83 and 1454.00 (2 × C-O-); **^1^H NMR** (δ, ppm): 5.28 (2H, t, *J* = 3.2 Hz, 2 × C_12_-H), 4.49 (2H, dd, *J* = 9.5 and 4.8 Hz, 2 × C_3_-H_α_), 4.04–3.96 (4H, m, -O-CH_2_-CH_2_-CH_2_-CH_2_-CH_2_-CH_2_-CH_2_-CH_2_-CH_2_-CH_2_-CH_2_-CH_2_-O-), 2.87 (2H, dd, *J* = 13.7 and 3.9 Hz, 2 × C_18_-H_β_), 2.05 (6H, s, 2 × CH_3_COO-), 1.13, 0.93, 0.92, 0.90, 0.86, 0.85, 0.73 (7 × 6H, 7 × s, 14 × CH_3_ groups); **^13^C NMR** (δ, ppm): 177.73 (2 × C_q_, 2 × C-28), 170.90 (2 × C_q_, 2 × CH_3_COO-), 143.79 (2 × C_q_, 2 × C-13), 122.17 (2 × CH, 2 × C-12), 80.84 (2 × CH, 2 × C-3), 64.19 (2 × CH_2_, -O-CH_2_-CH_2_-CH_2_-CH_2_-CH_2_-CH_2_-CH_2_-CH_2_-CH_2_-CH_2_-CH_2_-CH_2_-O-), 46.60 (2 × C_q_, 2 × C-17), 29.60 (2 × CH_2_, -O-CH_2_-CH_2_-CH_2_-CH_2_-CH_2_-CH_2_-CH_2_-CH_2_-CH_2_-CH_2_-CH_2_-CH_2_-O-), 29.58 (2 × CH_2_, -O-CH_2_-CH_2_-CH_2_-CH_2_-CH_2_-CH_2_-CH_2_-CH_2_-CH_2_-CH_2_-CH_2_-CH_2_-O-), 29.20 (2 × CH_2_, -O-CH_2_-CH_2_-CH_2_-CH_2_-CH_2_-CH_2_-CH_2_-CH_2_-CH_2_-CH_2_-CH_2_-CH_2_-O-), 28.57 (2 × CH_2_, -O-CH_2_-CH_2_-CH_2_-CH_2_-CH_2_-CH_2_-CH_2_-CH_2_-CH_2_-CH_2_-CH_2_-CH_2_-O-), 27.99 (2 × CH_2_, -O-CH_2_-CH_2_-CH_2_-CH_2_-CH_2_-CH_2_-CH_2_-CH_2_-CH_2_-CH_2_-CH_2_-CH_2_-O-), 21.28 (2 × C_q_, 2 × CH_3_COO-); **DEPT:** CH_3_: 8 × 2 CH_3_ (8 signals, 16 CH_3_ groups), CH_2_: 16 × 2 CH_2_ (16 signals, 32 CH_2_ groups), CH: 5 × 2 CH (5 signals, 10 CH groups).

### 4.3. Polarity

The polarity of the obtained AcOADs (**3a**–**3n**) was assessed by the High-Performance Thin-Layer Chromatography (HP TLC) method based on the Rf value [41] and compared to that of the parent compound (**1**) and unsubstituted oleanolic acid dimers (OADs). In short, the dichloromethane solutions of AcOADs (**3a**–**3n**) were applied on the starting line of TLC plates. The plates were developed in a horizontal chamber with mixtures of benzene and ethyl acetate in various volume ratios. Next, the plates were dried, the spots were visualized, and finally, the R_f_ values were calculated. The details of the experiments are given in [41].

### 4.4. SAR Study

The Structure–Activity Relationship Analysis was performed with the application of the PASS (Prediction of Activity Spectra for Substance) computer system [45]. This system predicts many types of pharmacological activity and mechanisms of action based on the structure of a compound, using for this purpose appropriate MNA (Multilevel Neighborhoods of Atoms) descriptors. As a result of such mathematical analysis, the prediction result is obtained in the form of a list of found types of activities. The probability of occurrence of a given activity is defined as P_a_, and the probability of a given activity not occurring as P_i_. Both values are expressed in a range between 0 and 1.

### 4.5. MTT Assay

The MTT assay was conducted as described earlier, e.g., [44]. In short, this method is based on the reaction between mitochondria enzymes (dehydrogenases) of tested cell lines and 3-(4,5-dimethylthiazol-2-yl)-2,5-diphenyltetrazolium bromide (MTT). The final product of this reaction gives violet formazan crystals. The number of live cells is proportional to the amount of formazan and is represented by the violet dye reagent. The details of the experiments are given in [44].

### 4.6. Antioxidant Activity

The DPPH assay relies on the ability of an antioxidant to donate a hydrogen atom or an electron to the stable DPPH radical, resulting in its reduction and subsequent color change. In contrast, the CUPRAC method measures the reducing power of a sample by quantifying its ability to convert the Cu-neocuproine reagent complex into the Cu(I) form. The results are presented as % inhibition of the copper(II) ions and Trolox equivalent (blue bars) calculated from the standard curve (Supplementary Materails S02, Figure S1) and as % inhibition of the DPPH radical and Trolox equivalent (pink bars), calculated from the standard curve (Supplementary Materials S02, Figure S2). The details of the experiments are given in [46].

## 5. Conclusions

Among numerous methods that have been developed for the transformation of oleanolic acid and other triterpenes into derivatives with a high level of pharmacological activity, particular attention should be paid to the effects of dimerization. It is an economic operation in every respect—no long reaction time, no heating or complicated applications are required, no expensive, hard-to-access, or dangerous chemical reagents are applied, and properly defined conditions of reaction provide products with a very high level of purity.

As a result of a properly designed and planned acetylation reaction, economical and safe, 14 Acetylated Oleanolic Acid Dimers (AcOADs, Figure 2), unknown in the scientific literature, were obtained.

The disubstituted dimers were crystallized using two methods, and their melting points were compared, plotting them in the form of graphs (Figure 4 and Figure 5). The structure of the newly obtained AcOADs **3a**–**3n** was proven beyond any doubt based on spectral data (Table 5, Table 6 and Table 7).

All obtained AcOADs **3a**–**3n** were less polar compounds than their mother compound, oleanolic acid (**1**) (Table 4), which is due to the blocking of the polar hydroxyl group in the C-3 position and the polar carboxyl function at the C-17 position of both triterpene units.

The Structure–Activity Relationship (SAR) analysis provided insights into the pharmacological potential of the synthesized AcOADs **3a**–**3n** (Table 1 and Table 2). Key findings from the SAR analysis include antiprotozoal, apoptosis agonist, caspase stimulation, chemopreventive, and hepatoprotective activities.

The MTT test presented high cytotoxicity of the obtained AcOADs towards all four cancer cell lines, with several compounds achieving IC_50_ values well below 5.00 µM (Table 3). The results also indicated high selectivity of most of the tested dimers **3a**–**3n** towards cancer cells (Table 3). Five of the fourteen disubstituted **3a**–**3n** dimers tested, i.e., AcOADs **3a**, **3b**, **3e**, **3j**, and **3m** (with a one-, two-, eight- or eleven-carbon linker, respectively), have a chance to become potential anticancer compounds because their Selectivity Index exceeded 2, while for dimer **3a**, with the shortest bridge, was even approximately 3 (Table 3). Additionally, in the DPPH assay, AcOADs showed significant free radical scavenging ability, with some derivatives achieving Trolox equivalent above 0.04 mg/mL (Figure 3).

The synthesized AcOADs exhibit significant promise as both cytostatic and antioxidant agents. Their ability to selectively target and kill cancer cells, coupled with high antioxidant activity, positions them as potential therapeutic agents for treating various cancers and oxidative stress-related conditions. The SAR analysis supports these findings, suggesting that specific structural modifications can further enhance their efficacy and selectivity. Continued research and development of these compounds could lead to new, effective treatments for cancer and other diseases associated with oxidative stress.

## Figures and Tables

**Table 4:** The comparison of R_f_ values of oleanolic acid (**1**), acetyloleanolic acid (AcAO), and AcOADs **3a**–**3n**.

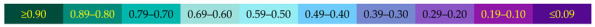									

**Number of C Atoms** **in a Linker**	**Comp. No.**	**R_f_ value in C_6_H_6_: AcOEt (*v:v*)**							
		**AcOEt**	**1:1**	**2:1**	**4:1**	**9:1**	**15:1**	**25:1**	**C_6_H_6_**
**0**	**1 (OA)**	0.86	0.77	0.62	0.29	0.16	---	---	---
**0**	**Ac-OA**	---	---	---	0.80	0.57	0.44	0.30	0.06
**1**	**3a**	---	---	---	0.94	0.82	0.71	0.61	0.28
**2 sat.**	**3b**	---	---	---	0.93	0.79	0.69	0.54	0.17
**3 sat.**	**3c**	---	---	---	0.93	0.80	0.69	0.55	0.17
**4 sat.**	**3d**	---	---	---	0.93	0.79	0.68	0.54	0.12
**4 unsat. *cis***	**3e**	---	---	---	0.93	0.81	0.70	0.57	0.16
**4 unsat. *trans***	**3f**	---	---	---	0.93	0.80	0.69	0.55	0.14
**5 sat.**	**3g**	---	---	---	0.93	0.80	0.68	0.55	0.14
**6 sat.**	**3h**	---	---	---	0.93	0.79	0.67	0.55	0.14
**7 sat.**	**3i**	---	---	---	0.94	0.81	0.68	0.55	0.14
**8 sat.**	**3j**	---	---	---	0.95	0.81	0.69	0.55	0.14
**9 sat.**	**3k**	---	---	---	0.95	0.82	0.69	0.56	0.14
**10 sat.**	**3l**	---	---	---	0.95	0.82	0.70	0.57	0.14
**11 sat.**	**3m**	---	---	---	0.95	0.83	0.70	0.58	0.14
**12 sat.**	**3n**	---	---	---	0.95	0.84	0.71	0.58	0.14

**Legend**: **OA**—oleanolic acid (reference compound); **sat.**—saturated linker; **unsat.**—unsaturated linker; **Ac-OA**—acetyloleanolic acid; **R_f_**—retention factor; ***v:v***—volume ratio; **AcOEt**—ethyl acetate; **C_6_H_6_**—benzene.

**Table 5:** The comparison of wavenumber values (ν) for absorption bands derived from the most characteristic functional groups within molecules of the dimers and **3a**–**3n**.

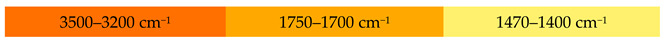				

**Number of C Atoms** **in a Linker**	**Comp. No.**	**ν [cm^−1^]**		
		**OH**	**C=O**	**C-O-**
**0**	**1**	3446 *	1687 *	1452 *
**1**	**3a**	---	1730.55, 1718.66	1461.76, 1454.02
**2 sat.**	**3b**	---	1730.49, 1718.46	1461.95, 1453.93
**3 sat.**	**3c**	---	1731.53, 1718.88	1462.72, 1454.82
**4 sat.**	**3d**	---	1730.30, 1718.02	1462.19, 1453.54
**4 unsat. *cis***	**3e**	---	1729.25	1463.43, 1453.40
**4 unsat. *trans***	**3f**	---	1731.30, 1719.34	1464.58, 1456.30
**5 sat.**	**3g**	---	1728.96, 1718.50	1463.13, 1454.76
**6 sat.**	**3h**	---	1730.48, 1717.67	1463.84, 1454.52
**7 sat.**	**3i**	---	1729.19, 1718.95	1463.99, 1453.76
**8 sat.**	**3j**	---	1729.02, 1718.44	1464.02, 1453.33
**9 sat.**	**3k**	---	1728.66, 1720.10	1463.55, 1454.11
**10 sat.**	**3l**	---	1730.90, 1717.99	1464.91, 1453.72
**11 sat.**	**3m**	---	1729.09, 1718.18	1464.55, 1453.66
**12 sat.**	**3n**	---	1729.16, 1718.24	1463.83, 1454.00

**Legend**: **OA**—oleanolic acid (reference compound); **ν**—wavenumber value; **sat.**—saturated linker; **unsat.**—unsaturated linker; *—published in [49].

**Table 6:** The comparison of the most characteristic signals from ^1^H NMR spectra for oleanolic acid (**1**) and AcOADs (**3a**–**3n**).

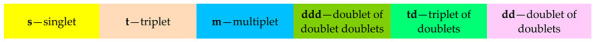						

**Number** **of C Atoms** **in a Linker**	**Comp. No.**	**Chemical Shift, δ [ppm] (Multiplicity, *J* [Hz])**				
		**C_12_-H**	**Linker**	**C_3_-H_α_**	**C_18_-H_β_**	**CH_3_COO-**
**0**	**1 (OA)**	5.27 (t, n.d) *	---	3.18 (dd, 11.0, 5.0) *	2.85 (dd, 14.0, 4.0) *	---
**1**	**3a**	5.28 (t, 3.4)	5.73 (s)	4.48 (dd, 9.4, 4.8)	2.82 (dd, 13.5, 3.5)	2.03 (s)
**2 sat.**	**3b**	5.29 (t, 3.2)	4.30–4.08 (m)	4.49 (dd, 8.4, 7.2)	2.86 (dd, 13.2, 3.7)	2.05 (s)
**3 sat.**	**3c**	5.28 (t, 3.3)	4.09 (t, 6.3)	4.49 (dd, 9.7, 7.9)	2.85 (dd, 13.6, 3.9)	2.04 (s)
**4 sat.**	**3d**	5.28 (t, 3.3)	4.09–3.98 (m)	4.49 (dd, 9.7, 7.9)	2.86 (dd, 13.6, 3.8)	2.05 (s)
**4 unsat. *cis***	**3e**	5.29 (t, 3.5)	4.63 (ddd, 17.9, 13.0, 4.4)	4.49 (dd, 9.5, 7.5)	2.85 (dd, 13.1, 4.4)	2.06 (s)
**4 unsat. *trans***	**3f**	5.30 (t, 3.2)	4.53 (t, 13.9)	4.49 (dd, 9.5, 7.5)	2.88 (dd, 13.7, 3.9)	2.06 (s)
**5 sat.**	**3g**	5.28 (t, 3.2)	4.02 (t, 6.4)	4.49 (dd, 7.9, 9.2)	2.86 (dd, 13.8, 4.0)	2.05 (s)
**6 sat.**	**3h**	5.29 (t, 3.4)	4.02 (t, 6.5)	4.50 (dd, 7.9, 4.4)	2.87 (dd, 14.1, 4.1)	2.06 (s)
**7 sat.**	**3i**	5.28 (t, 3.4)	4.05–3.97 (m)	4.50 (dd 7.9, 4.5)	2.88 (dd, 13.8, 4.0)	2.06 (s)
**8 sat.**	**3j**	5.27 (t, 3.2)	4.07–3.94 (m)	4.50 (dd, 7.9, 4.5)	2.86 (dd, 13.9, 3.5)	2.04 (s)
**9 sat.**	**3k**	5.28 (t, 3.2)	4.15–3.94 (m)	4.50 (dd, 7.9, 4.4)	2.88 (dd, 13.6, 3.9)	2.05 (s)
**10 sat.**	**3l**	5.25 (t, 3.4)	4.08–3.94 (m)	4.49 (dd, 10.2, 8.7)	2.88 (dd, 14.4, 3.2)	2.03 (s)
**11 sat.**	**3m**	5.27 (t, 3.7)	4.00 (td, 6.4, 3.1)	4.49 (dd, 10.1, 8.7)	2.87 (dd, 13.3, 4.6)	2.05 (s)
**12 sat.**	**3n**	5.28 (t, 3.2)	4.04–3.96 (m)	4.49 (dd, 9.5, 4.8)	2.87 (dd, 13.7, 3.9)	2.05 (s)

**Legend**: **OA**—oleanolic acid (reference compound); **δ**—chemical shift; **sat.**—saturated linker; **unsat.**—unsaturated linker; **n.d.**—no data, ***J***—coupling constant, ***Hz***—hertz (the unit of frequency); *—published in [49].

**Table 7:** The comparison of the most characteristic signals from ^13^C NMR spectra of the dimers **3a**–**3n**.

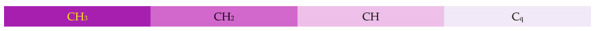									

**Number of C Atoms in a Linker**	**Comp. No.**	**Chemical Shift, δ [ppm]**							
		**C-28**	**CH_3_COO-**	**C-13**	**C-12**	**C-3**	**linker**	**C-17**	**CH_3_COO-**
**0**	**1 (OA)**	180.40 *	---	143.79 *	122.25 *	78.31 *	---	45.85 *	---
**1**	**3a**	176.31	171.03	143.35	122.54	80.90	79.36	46.75	21.29
**2 sat.**	**3b**	177.40	170.94	143.54	122.38	80.83	62.14	46.66	21.27
**3 sat.**	**3c**	177.45	170.92	143.63	122.26	80.78	60.74	46.64	21.25
**4 sat.**	**3d**	177.59	170.95	143.72	122.23	80.80	63.62	46.63	21.28
**4 unsat. *cis***	**3e**	177.28	170.97	143.60	122.29	80.84	59.60	46.60	21.27
**4 unsat. *trans***	**3f**	177.30	171.02	143.67	122.36	80.88	63.70	46.75	21.31
**5 sat.**	**3g**	177.64	170.96	143.74	122.22	80.83	63.93	46.61	21.27
**6 sat.**	**3h**	177.59	170.97	143.80	122.21	80.86	64.03	46.64	21.27
**7 sat.**	**3i**	177.74	171.02	143.83	122.24	80.90	64.15	46.67	21.31
**8 sat.**	**3j**	177.73	171.02	143.81	122.22	80.90	64.19	46.65	21.30
**9 sat.**	**3k**	177.74	170.98	143.86	122.24	80.89	64.21	46.66	21.31
**10 sat.**	**3l**	177.73	170.97	143.83	122.22	80.88	64.21	46.65	21.30
**11 sat.**	**3m**	177.72	170.93	143.83	122.21	80.87	64.20	46.65	21.30
**12 sat.**	**3n**	177.73	170.90	143.79	122.17	80.84	64.19	46.60	21.28

**Legend**: **OA**—oleanolic acid (reference compound); **δ**—chemical shift; **sat.**—saturated linker; **unsat.**—unsaturated linker; *—published in [49].

## Data Availability

All data concerning this paper are available in the manuscript body or the Supplementary Data.

## Supplementary Materials

The following supporting information can be downloaded at: https://www.mdpi.com/article/10.3390/molecules29184291/s1, Supplementary Materals S01. Table S1. The predicted activity of oleanolic acid (1) and its derivatives 3a–3n determined by the PASS method. Supplementary Materals S02. Figure S1. Standard curve for CUPRAC radical inhibition by Trolox. Figure S2. Standard curve for DPPH radical inhibition by Trolox. Supplementary Materals S03. NMR spectra of dimer 3a.
