# Supplementary material for: Acetylation of Oleanolic Acid Dimers as a Method of Synthesis of Powerful Cytotoxic Agents
Source: Molecules. 2024 Sep 10;29(18):4291. doi: 10.3390/molecules29184291 (PMC11434080; doi:10.3390/molecules29184291)
Supplement: Supplementary file 1 [file molecules-29-04291-s001.zip › molecules-3163429-supplementary.pdf]

Article

Acetylation of Oleanolic Acid Dimers as a Method of Synthesis of Powerful Cytotoxic Agents

Andrzej Günther, Przemysław Zalewski, Szymon Sip, Piotr Ruszkowski and Barbara Bednarczyk-Cwynar

Supplementary Materials S01

Table S1. Predicted activity of oleanolic acid (**1**) and its derivatives **3a** – **3n** determined by the PASS method.

1.000 – 0.950	0.949 – 0.900	0.899 – 0.850	0.849 – 0.800	0.799 – 0.750	0.749 – 0.700	< 0.700

	activity	P_a factor (and P_i factor) of compounds 1 and 3a – 3n (P_a > 0.700)														
		OA (1)	3a	3b	3c	3d	3e	3f	3g	3h	3i	3j	3k	3l	3m	3n
1	**Acylcarnitine hydrolase inhibitor**	*0.711 (0.025)*	< 0.700	< 0.700	< 0.700	< 0.700	< 0.700	< 0.700	< 0.700	< 0.700	< 0.700	< 0.700	< 0.700	< 0.700	< 0.700	< 0.700
2	**AGPCh hydrolase inhibitor**	*0.825 (0.014)*	< 0.700	< 0.700	< 0.700	< 0.700	< 0.700	< 0.700	< 0.700	< 0.700	< 0.700	< 0.700	< 0.700	< 0.700	< 0.700	< 0.700
3	**Antiinflammatory**	*0.819 (0.005)*	0.862 (0.005)	0.811 (0.006)	0.807 (0.006)	0.804 (0.006)	0.773 (0.009)	0.773 (0.009)	0.802 (0.007)	0.802 (0.007)	0.802 (0.007)	0.802 (0.007)	0.802 (0.007)	0.802 (0.007)	0.802 (0.007)	0.802 (0.007)
4	**Antineoplastic**	*0.876 (0.005)*	0.893 (0.005)	0.886 (0.005)	0.862 (0.006)	0.866 (0.005)	0.881 (0.005)	0.881 (0.005)	0.861 (0.006)	0.861 (0.006)	0.861 (0.006)	0.861 (0.006)	0.861 (0.006)	0.861 (0.006)	0.861 (0.006)	0.861 (0.006)
5	**Antineoplastic (colon cancer)**	*< 0.700*	0.734 (0.005)	< 0.700	< 0.700	< 0.700	< 0.700	< 0.700	< 0.700	< 0.700	< 0.700	< 0.700	< 0.700	< 0.700	< 0.700	< 0.700
6	**Antineoplastic (colorectal cancer)**	*< 0.700*	0.732 (0.005)	< 0.700	< 0.700	< 0.700	< 0.700	< 0.700	< 0.700	< 0.700	< 0.700	< 0.700	< 0.700	< 0.700	< 0.700	< 0.700
7	**Antineoplastic (lung cancer)**	*0.766 (0.005)*	0.801 (0.004)	0.792 (0.004)	0.773 (0.005)	0.777 (0.004)	0.775 (0.004)	0.775 (0.004)	0.769 (0.005)	0.769 (0.005)	0.769 (0.005)	0.769 (0.005)	0.769 (0.005)	0.769 (0.005)	0.769 (0.005)	0.769 (0.005)
8	**Antinociceptive**	*< 0.700*	0.787 (0.002)	0.804 (0.002)	0.786 (0.002)	0.786 (0.002)	0.791 (0.002)	0.791 (0.002)	0.788 (0.002)	0.788 (0.002)	0.788 (0.002)	0.788 (0.002)	0.788 (0.002)	0.788 (0.002)	0.788 (0.002)	0.788 (0.002)
9	**Antiprotozoal (Leishmania)**	*0.721 (0.008)*	0.820 (0.004)	0.862 (0.004)	0.892 (0.003)	0.891 (0.003)	0.854 (0.004)	0.854 (0.004)	0.904 (0.003)	0.904 (0.003)	0.904 (0.003)	0.904 (0.003)	0.904 (0.003)	0.904 (0.003)	0.904 (0.003)	0.904 (0.003)

10	**Antipruritic**	*0.776 (0.004)*	< 0.700	0.712 (0.007)	0.726 (0.006)	0.759 (0.005)	< 0.700	< 0.700	0.766 (0.004)	0.766 (0.004)	0.766 (0.004)	0.766 (0.004)	0.766 (0.004)	0.766 (0.004)	0.766 (0.004)	0.766 (0.004)
11	**Antisecretoric**	*0.707 (0.009)*	0.756 (0.005)	0.869 (0.003)	0.850 (0.004)	0.874 (0.003)	0.872 (0.003)	0.872 (0.003)	0.884 (0.003)	0.884 (0.003)	0.884 (0.003)	0.884 (0.003)	0.884 (0.003)	0.884 (0.003)	0.884 (0.003)	0.884 (0.003)
12	**Antitussive**	*< 0.700*	0.816 (0.003)	0.795 (0.003)	0.730 (0.004)	0.768 (0.004)	< 0.700	< 0.700	0.735 (0.004)	0.735 (0.004)	0.735 (0.004)	0.735 (0.004)	0.735 (0.004)	0.735 (0.004)	0.735 (0.004)	0.735 (0.004)
13	**Antiviral (Influenza)**	*0.836 (0.002)*	< 0.700	0.735 (0.004)	0.712 (0.005)	0.736 (0.004)	0.720 (0.005)	0.720 (0.005)	0.742 (0.004)	0.742 (0.004)	0.742 (0.004)	0.742 (0.004)	0.742 (0.004)	0.742 (0.004)	0.742 (0.004)	0.742 (0.004)
14	**Antiulcerative**	*0.771 (0.004)*	< 0.700	0.818 (0.004)	0.822 (0.003)	0.816 (0.004)	0.867 (0.003)	0.867 (0.003)	0.825 (0.003)	0.825 (0.003)	0.825 (0.003)	0.825 (0.003)	0.825 (0.003)	0.866 (0.005)	0.866 (0.005)	0.866 (0.005)
15	**Apoptosis agonist**	*0.901 (0.004)*	0.905 (0.004)	0.881 (0.005)	0.866 (0.005)	0.868 (0.005)	0.874 (0.005)	0.874 (0.005)	0.866 (0.005)	0.866 (0.005)	0.866 (0.005)	0.866 (0.005)	0.866 (0.005)	0.866 (0.005)	0.866 (0.005)	0.866 (0.005)
16	**Caspase 3 stimulant**	*0.984 (0.002)*	0.904 (0.003)	0.950 (0.003)	0.909 (0.003)	0.909 (0.003)	0.902 (0.003)	0.902 (0.003)	0.910 (0.003)	0.910 (0.003)	0.910 (0.003)	0.910 (0.003)	0.910 (0.003)	0.910 (0.003)	0.910 (0.003)	0.910 (0.003)
17	**Caspase 8 stimulant**	*0.914 (0.001)*	0.861 (0.001)	0.889 (0.001)	0.873 (0.001)	0.873 (0.001)	0.852 (0.001)	0.852 (0.001)	0.879 (0.001)	0.879 (0.001)	0.879 (0.001)	0.879 (0.001)	0.879 (0.001)	0.879 (0.001)	0.879 (0.001)	0.879 (0.001)
18	**Chemopreventive**	*0.937 (0.002)*	0.915 (0.002)	0.896 (0.002)	0.881 (0.003)	0.888 (0.003)	0.928 (0.002)	0.928 (0.002)	0.888 (0.003)	0.888 (0.003)	0.888 (0.003)	0.888 (0.003)	0.888 (0.003)	0.888 (0.003)	0.888 (0.003)	0.888 (0.003)
19	**Chitinase inhibitor**	*0.789 (0.003)*	< 0.700	< 0.700	< 0.700	< 0.700	< 0.700	< 0.700	< 0.700	< 0.700	< 0.700	< 0.700	< 0.700	< 0.700	< 0.700	< 0.700
20	**Cholesterol antagonist**	*< 0.700*	< 0.700	< 0.700	< 0.700	< 0.700	< 0.700	< 0.700	0.705 (0.008)	0.705 (0.008)	0.705 (0.008)	0.705 (0.008)	0.705 (0.008)	0.705 (0.008)	0.705 (0.008)	0.705 (0.008)
21	**CYP2J substrate**	*0.808 (0.019)*	< 0.700	< 0.700	< 0.700	< 0.700	< 0.700	< 0.700	< 0.700	< 0.700	< 0.700	< 0.700	< 0.700	< 0.700	< 0.700	< 0.700
22	**Cytoprotectant**	*0.709 (0.004)*	< 0.700	0.701 (0.005)	< 0.700	0.708 (0.005)	0.755 (0.003)	0.755 (0.003)	0.714 (0.004)	0.714 (0.004)	0.714 (0.004)	0.714 (0.004)	0.714 (0.004)	0.714 (0.004)	0.714 (0.004)	< 0.700
23	**DNA ligase (ATP) inhibitor**	*0.803 (0.001)*	< 0.700	< 0.700	< 0.700	< 0.700	< 0.700	< 0.700	< 0.700	< 0.700	< 0.700	< 0.700	< 0.700	< 0.700	< 0.700	< 0.700
24	**Gastrin inhibitor**	*< 0.700*	< 0.700	< 0.700	0.735 (0.004)	0.734 (0.004)	< 0.700	< 0.700	0.743 (0.003)	0.743 (0.003)	0.743 (0.003)	0.743 (0.003)	0.743 (0.003)	0.743 (0.003)	0.743 (0.003)	0.743 (0.003)
25	**Hepatic disorders treatment**	*0.834 (0.004)*	0.784 (0.004)	0.801 (0.004)	0.785 (0.004)	0.797 (0.004)	0.805 (0.004)	0.805 (0.004)	0.790 (0.004)	0.790 (0.004)	0.790 (0.004)	0.790 (0.004)	0.790 (0.004)	0.790 (0.004)	0.790 (0.004)	0.790 (0.004)
26	**Hepatoprotectant**	*0.930 (0.002)*	0.915 (0.002)	0.939 (0.002)	0.941 (0.002)	0.958 (0.001)	0.959 (0.001)	0.959 (0.001)	0.963 (0.001)	0.963 (0.001)	0.963 (0.001)	0.963 (0.001)	0.963 (0.001)	0.963 (0.001)	0.963 (0.001)	0.963 (0.001)
27	**Hypolipemic**	*0.713 (0.013)*	0.788 (0.008)	0.837 (0.006)	0.862 (0.005)	0.857 (0.005)	0.872 (0.005)	0.872 (0.005)	0.871 (0.005)	0.871 (0.005)	0.871 (0.005)	0.871 (0.005)	0.871 (0.005)	0.871 (0.005)	0.871 (0.005)	0.871 (0.005)
28	**ICAM1 expression**	*0.747*	< 0.700	< 0.700)	< 0.700	< 0.700	< 0.700	< 0.700	< 0.700	< 0.700	< 0.700	< 0.700	< 0.700	< 0.700	< 0.700	< 0.700

	**inhibitor**	*(0.001)*														
29	**Insulin promotor**	*0.869 (0.004)*	0.881 (0.003)	0.941 (0.002)	0.928 (0.002)	0.928 (0.002)	0.920 (0.003)	0.920 (0.003)	0.922 (0.002)	0.922 (0.002)	0.922 (0.002)	0.922 (0.002)	0.922 (0.002)	0.922 (0.002)	0.922 (0.002)	0.922 (0.002)
30	**Lipid metabolism regulator**	*< 0.700*	0.822 (0.005)	0.959 (0.002)	0.949 (0.003)	0.959 (0.002)	0.960 (0.002)	0.960 (0.002)	0.963 (0.002)	0.963 (0.002)	0.963 (0.002)	0.963 (0.002)	0.963 (0.002)	0.963 (0.002)	0.963 (0.002)	0.963 (0.002)
31	**Lipid peroxidase inhibitor**	*0.810 (0.003)*	0.804 (0.003)	0.713 (0.005)	0.714 (0.005)	0.720 (0.005)	< 0.700	< 0.700	0.738 (0.004)	0.738 (0.004)	0.738 (0.004)	0.738 (0.004)	0.738 (0.004)	0.738 (0.004)	0.738 (0.004)	0.738 (0.004)
32	**Membrane integrity antagonist**	*0.928 (0.002)*	0.895 (0.003)	0.936 (0.001)	0.930 (0.002)	0.939 (0.001)	0.913 (0.002)	0.913 (0.002)	0.943 (0.001)	0.943 (0.001)	0.943 (0.001)	0.943 (0.001)	0.943 (0.001)	0.943 (0.001)	0.943 (0.001)	0.943 (0.001)
33	**Mucomembranous protectant**	*0.894 (0.005)*	0.826 (0.013)	0.873 (0.006)	0.850 (0.009)	0.850 (0.009)	0.863 (0.007)	0.863 (0.007)	0.857 (0.008)	0.857 (0.008)	0.857 (0.008)	0.857 (0.008)	0.857 (0.008)	0.857 (0.008)	0.857 (0.008)	0.857 (0.008)
34	**Nitric oxide antagonist**	*0.814 (0.002)*	0.798 (0.003)	0.787 (0.003)	0.764 (0.003)	0.770 (0.003)	0.772 (0.003)	0.772 (0.003)	0.762 (0.003)	0.762 (0.003)	0.762 (0.003)	0.762 (0.003)	0.762 (0.003)	0.762 (0.003)	0.762 (0.003)	0.762 (0.003)
35	**Oxidoreductase inhibitor**	*0.904 (0.002)*	0.875 (0.003)	0.904 (0.002)	0.897 (0.002)	0.904 (0.002)	0.913 (0.002)	0.913 (0.002)	0.907 (0.002)	0.907 (0.002)	0.907 (0.002)	0.907 (0.002)	0.907 (0.002)	0.907 (0.002)	0.907 (0.002)	0.907 (0.002)
36	**Phosphatase inhibitor**	*0.894 (0.001)*	< 0.700	< 0.700	< 0.700	< 0.700	< 0.700	< 0.700	< 0.700	< 0.700	< 0.700	< 0.700	< 0.700	< 0.700	< 0.700	< 0.700
37	**Protein phosphatase inhibitor**	*0.782 (0.002)*	< 0.700	< 0.700	< 0.700	< 0.700	< 0.700	< 0.700	< 0.700	< 0.700	< 0.700	< 0.700	< 0.700	< 0.700	< 0.700	< 0.700
38	**PT phosphatase inhibitor**	*0.764 (0.002)*	< 0.700	< 0.700	< 0.700	< 0.700	< 0.700	< 0.700	< 0.700	< 0.700	< 0.700	< 0.700	< 0.700	< 0.700	< 0.700	< 0.700
39	**T-cell PT phosphatase inhibitor**	*0.726 (0.001)*	< 0.700	< 0.700	< 0.700	< 0.700	< 0.700	< 0.700	< 0.700	< 0.700	< 0.700	< 0.700	< 0.700	< 0.700	< 0.700	< 0.700
40	**T-17β-dehydr. (NADP+) inhibitor**	*0.892 (0.007)*	< 0.700	< 0.700	< 0.700	< 0.700	< 0.700	< 0.700	< 0.700	< 0.700	< 0.700	< 0.700	< 0.700	< 0.700	< 0.700	< 0.700
41	**Transcription factor NF kappa B inhibitor**	*< 0.700*	< 0.700	0.733 (0.002)	0.720 (0.002)	0.738 (0.002)	0.715 (0.002)	0.715 (0.002)	0.732 (0.002)	0.732 (0.002)	0.732 (0.002)	0.732 (0.002)	0.732 (0.002)	0.732 (0.002)	0.732 (0.002)	0.732 (0.002)
42	**Transcription factor NF kappa B stimulant**	*0.954 (0.001)*	0.916 (0.001)	0.918 (0.001)	0.911 (0.001)	0.911 (0.001)	0.911 (0.001)	0.911 (0.001)	0.908 (0.001)	0.908 (0.001)	0.908 (0.001)	0.908 (0.001)	0.908 (0.001)	0.908 (0.001)	0.908 (0.001)	0.908 (0.001)
43	**Transcription factor stimulant**	*0.954 (0.001)*	0.916 (0.001)	0.918 (0.001)	0.911 (0.001)	0.911 (0.001)	0.911 (0.001)	0.911 (0.001)	0.908 (0.001)	0.908 (0.001)	0.908 (0.001)	0.908 (0.001)	0.908 (0.001)	0.908 (0.001)	0.908 (0.001)	0.908 (0.001)
44	**Vasodilator peripheral**	*< 0.700*	0.714 (0.008)	< 0.700	< 0.700	< 0.700	< 0.700	< 0.700	< 0.700	< 0.700	< 0.700	< 0.700	< 0.700	< 0.700	< 0.700	< 0.700

Legend: $\mathbf{P_a}$ = probability of activity; $\mathbf{P_i}$ = probability of inactivity; **AGPCh** = alkenylglycerophosphocholine; **DAGOA** = diacylglycerol *O*-acyl; **PT** = protein-tyrosine; **T-17β-dehydr.**= testosterone 17beta-dehydrogenase

Acetylation of Oleanolic Acid Dimers as a Method of Synthesis of Powerful Cytotoxic Agents

Andrzej Günther, Przemysław Zalewski, Szymon Sip, Piotr Ruszkowski and Barbara Bednarczyk-Cwynar

Supplementary Materials S02

Figure S1. Standard curve for CUPRAC radical inhibition by Trolox.

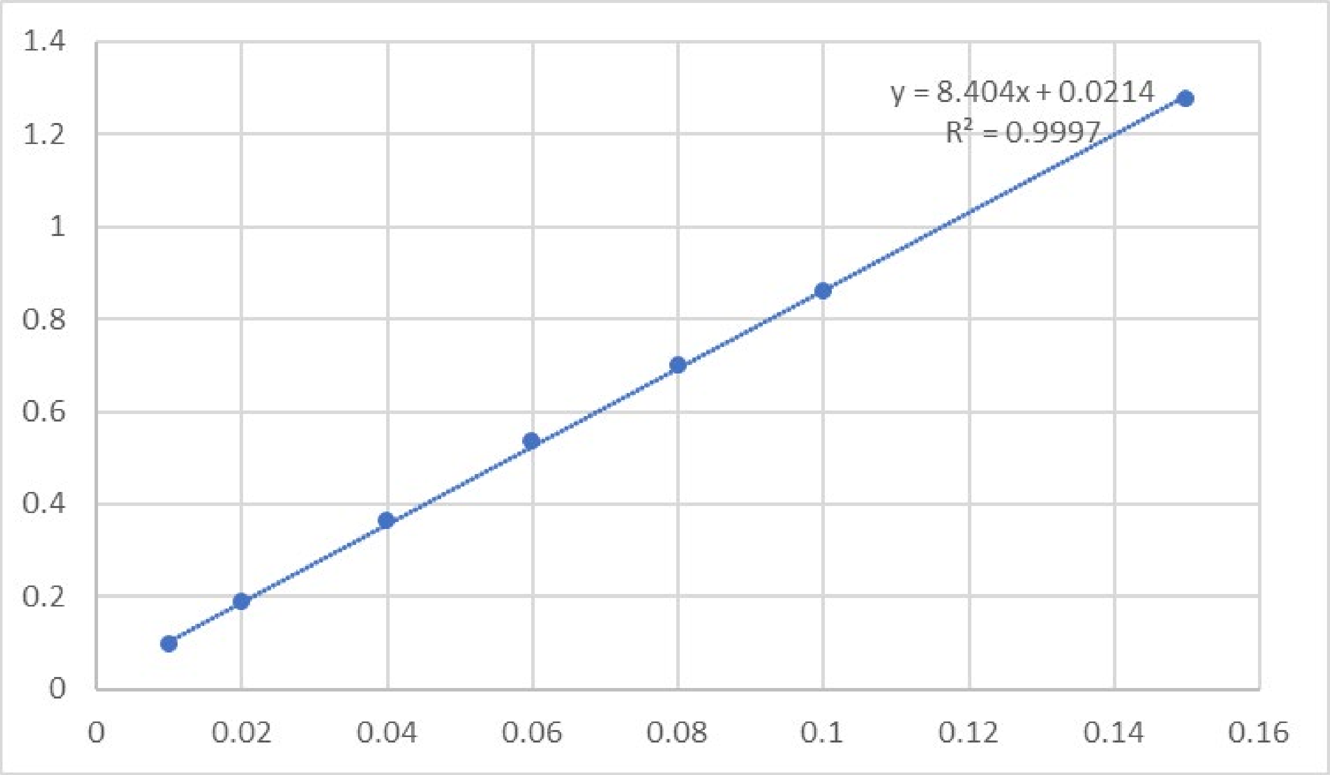

Figure 2. Standard curve for DPPH radical inhibition by Trolox.

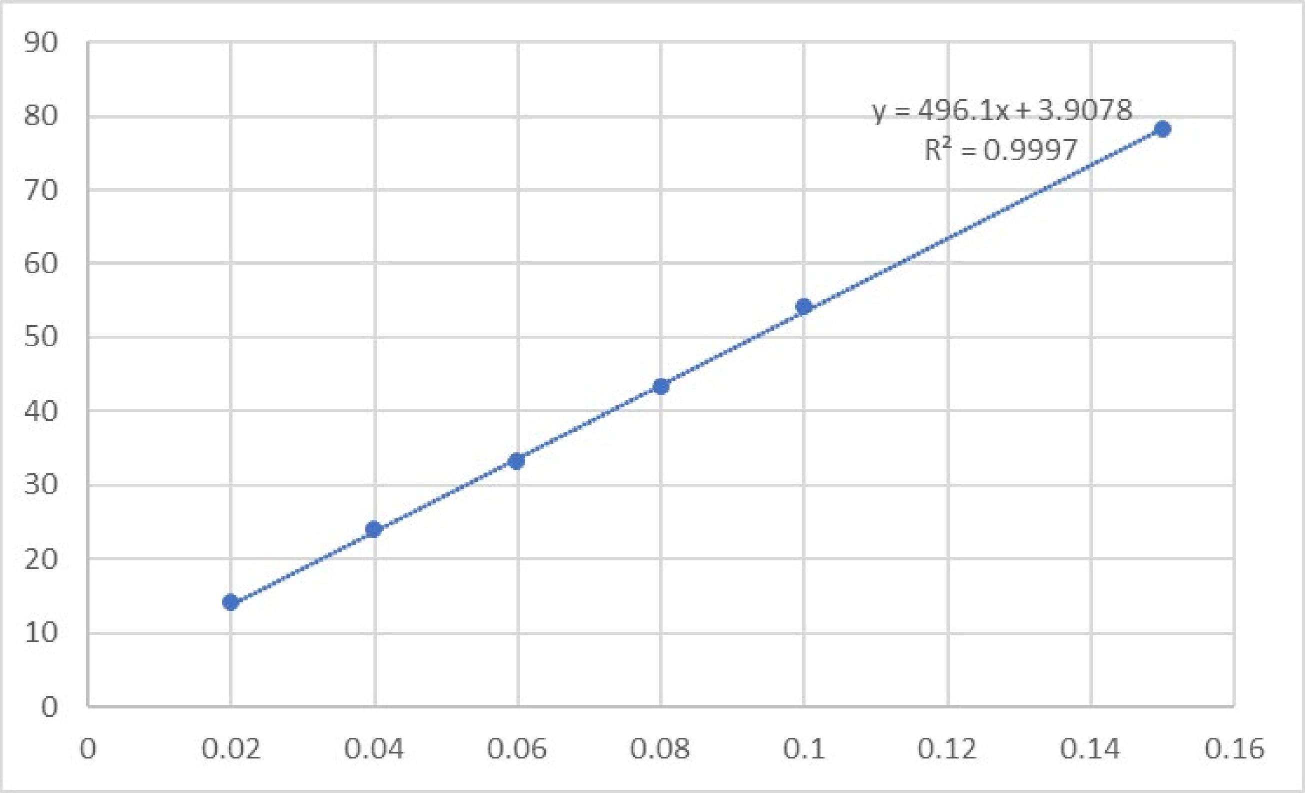

90
80
70
60
50
40
30
20
10
0
0
0.02
0.04
0.06
0.08
0.1
0.12
0.14
0.16
y = 496.1x + 3.9078
R² = 0.9997

Article

Acetylation of Oleanolic Acid Dimers as a Method of Synthesis of Powerful Cytotoxic Agents

Andrzej Günther, Przemysław Zalewski, Szymon Sip, Piotr Ruszkowski and Barbara Bednarczyk-Cwynar

Supplementary Materials S03

NMR spectra of dimer 3a

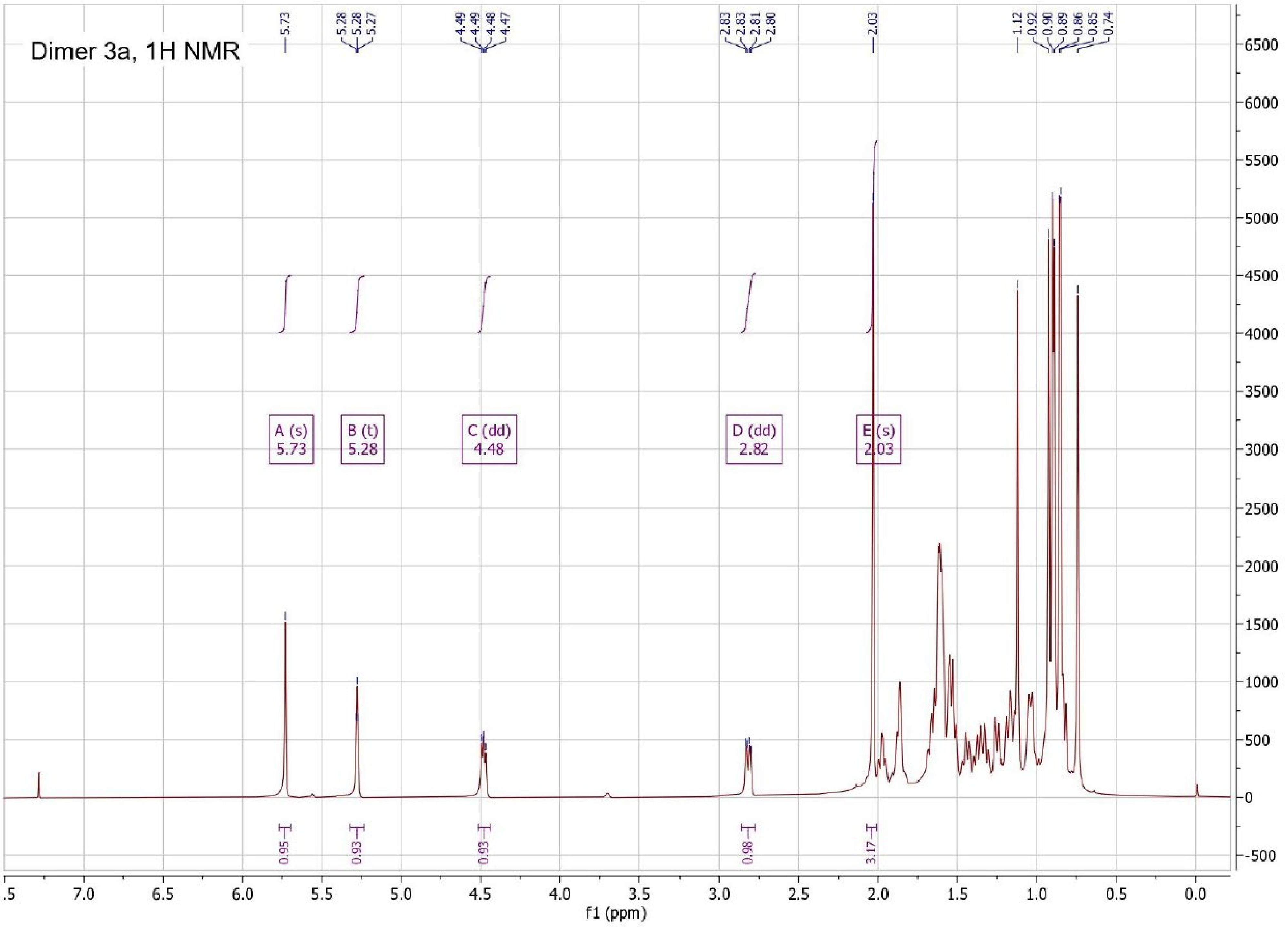
Dimer 3a, 1H NMR
5.73
5.28
5.28
5.27
4.49
4.49
4.48
4.47
2.83
2.83
2.81
2.80
2.03
1.12
0.92
0.90
0.89
0.86
0.85
0.74
A (s)
5.73
B (t)
5.28
C (dd)
4.48
D (dd)
2.82
E (s)
2.03
0.95
0.93
0.93
0.98
3.17
6500
6000
5500
5000
4500
4000
3500
3000
2500
2000
1500
1000
500
0
-500
.5
7.0
6.5
6.0
5.5
5.0
4.5
4.0
3.5
3.0
2.5
2.0
1.5
1.0
0.5
0.0
f1 (ppm)

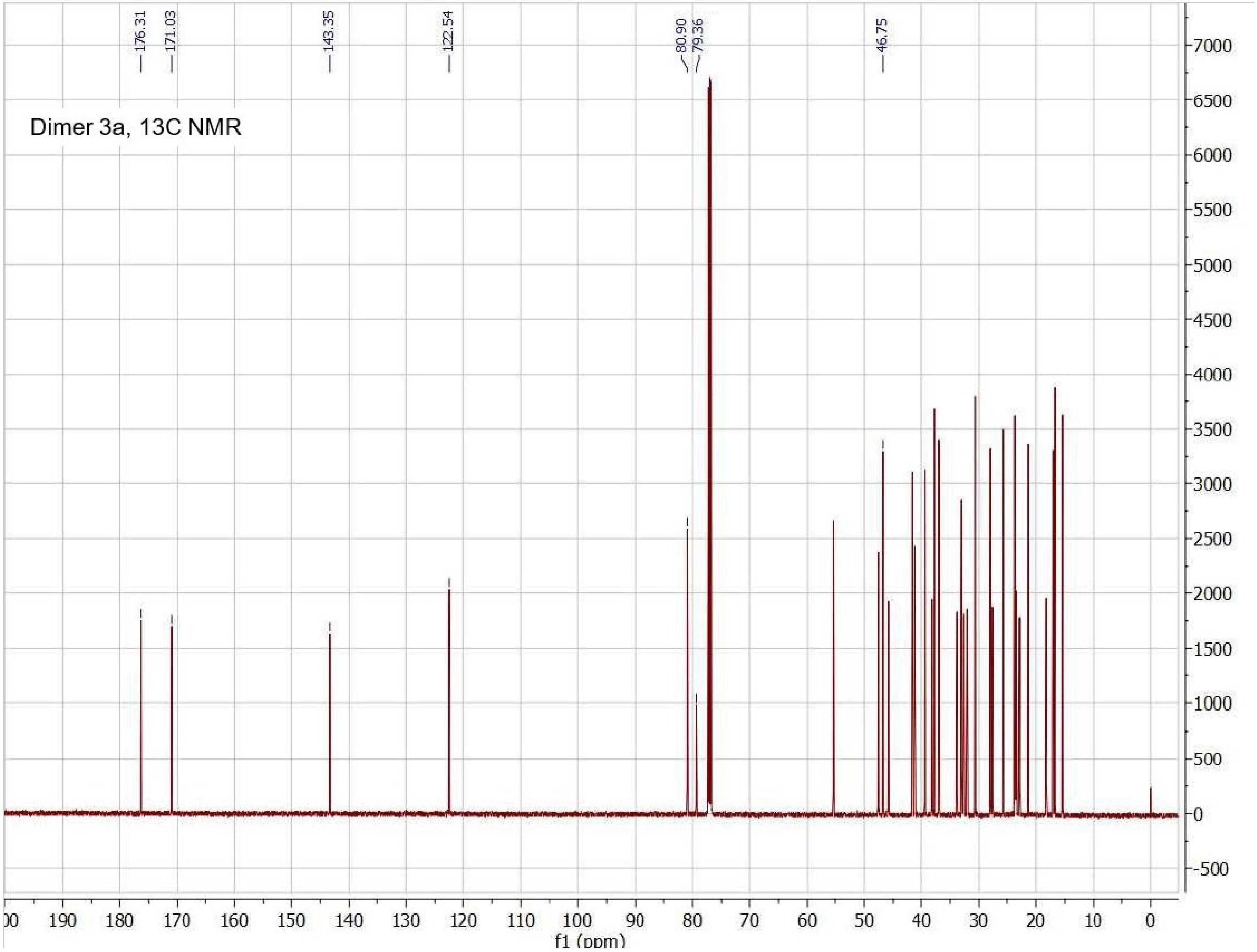
Dimer 3a, 13C NMR
176.31
171.03
143.35
122.54
80.90
79.36
46.75
f1 (ppm)

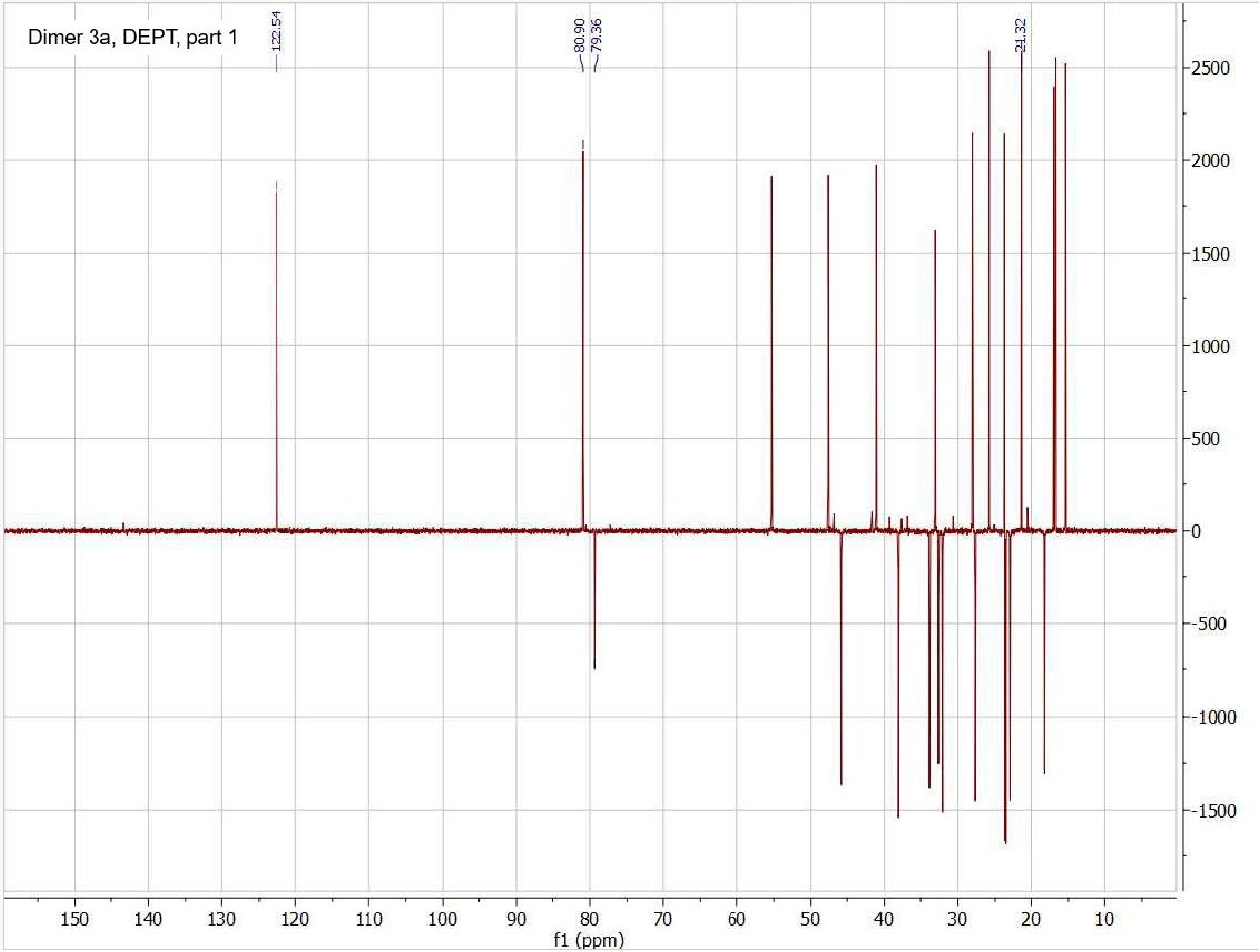
Dimer 3a, DEPT, part 1
122.54
80.90
79.36
21.32
2500
2000
1500
1000
500
0
-500
-1000
-1500
150
140
130
120
110
100
90
80
70
60
50
40
30
20
10
f1 (ppm)

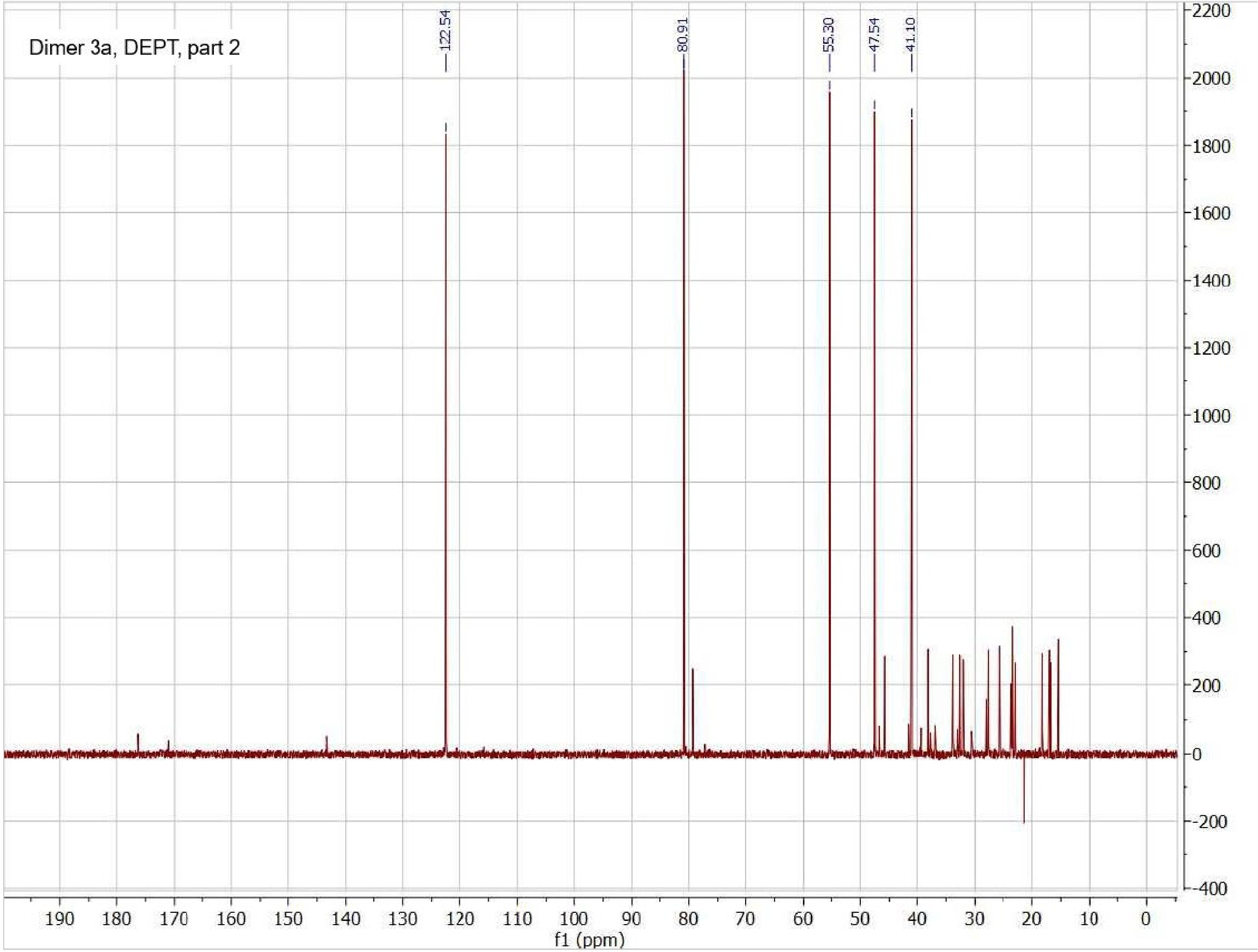

Dimer 3a, DEPT, part 2
122.54
80.91
55.30
47.54
41.10
2200
2000
1800
1600
1400
1200
1000
800
600
400
200
0
-200
-400
190
180
170
160
150
140
130
120
110
100
90
80
70
60
50
40
30
20
10
0
f1 (ppm)
